# Determination of the Thermal Parameters of Geopolymers Modified with Iron Powder

**DOI:** 10.3390/polym14102009

**Published:** 2022-05-13

**Authors:** Karol Prałat, Justyna Ciemnicka, Artur Koper, Michał Marek Szczypiński, Piotr Łoś, Van Vu Nguyen, Van Su Le, Cezary Rapiejko, Roberto Ercoli, Katarzyna Ewa Buczkowska

**Affiliations:** 1 Institute of Faculty of Civil Engineering, Faculty of Civil Engineering, Mechanics and Petrochemistry, Warsaw University of Technology, I. Łukasiewicza 17, 09-400 Płock, Poland; justyna.ciemnicka@pw.edu.pl (J.C.); artur.koper@pw.edu.pl (A.K.); 2 Department of Material Science, Faculty of Mechanical Engineering, Technical University od Liberec, Studentská 2, 461 17 Liberec, Czech Republic; michal.szczypinski@tul.cz (M.M.S.); piotr.los@tul.cz (P.Ł.); nguyen.van.vu@tul.cz (V.V.N.); su.le.van@tul.cz (V.S.L.); katarzyna.ewa.buczkowska@tul.cz (K.E.B.); 3 Department of Materials Technology and Production Systems, Faculty of Mechanical Engineering, Lodz University of Technology, 90-001 Lodz, Poland; cezary.rapiejko@p.lodz.pl; 4 Department of Pure and Applied Sciences, University of Urbino Carlo Bo, via Ca’ Le Suore 2/4, 61029 Urbino, Italy; r.ercoli@campus.uniurb.it

**Keywords:** micro additives, geopolymers, iron powder, thermal properties, thermal conductivity

## Abstract

The paper presents the results of research concerning the influence of a metallic micromaterial on the thermal conductivity *λ,* specific heat *Cp*, and thermal diffusivity *a* of modified geopolymers. Iron oxide in the form of powder with an average granulation of 10 μm was used as the geopolymer-modifying material. The research concerned geopolymer composite samples with metakaolin (activated with potassium silicate) and the addition of iron in amounts ranging from 0.5% to 2.5% in relation to the weight of the metakaolin. Additionally, the samples were modified with sand and fireclay in two different amounts—1:1 and 1:1.2 in relation to the metakaolin. The addition of fireclay caused a decrease in the thermal conductivity of the composites by 30% when compared to the samples with the addition of sand. The lowest value of the thermal conductivity coefficient *λ* was obtained for the geopolymer with metakaolin and fireclay. When the ratio of these components in the composite was 1:1, the value of thermal conductivity was equal to 0.6413 W/(m·K), while in the case of their ratio being 1:1.2, it was equal to 0.6456 W/(m·K). In the samples containing fireclay, no significant influence of the added iron on the values of thermal conductivity was noticed. In the case of the geopolymer with sand, the effect was noticeable, and it was most visible in the samples containing metakaolin and sand in the ratio of 1:1.2. It was noticed that with an increase in the addition of Fe, the thermal conductivity of the composite increased.

## 1. Introduction

The concept of ‘geopolymers’ was first introduced in the 1970s by the French scientist Joseph Davidovits, who defined them as inorganic polymeric materials with an amorphous, three-dimensional network structure [1]. They are produced by combining materials containing silica and alumina (e.g., metakaolin, fly ash, rice husk ash, volcanic tuff) with strong alkaline solutions [2,3,4,5,6], and can be defined as mineral polymers of geological origin that are formed in the process of geosynthesis. They consist of chains or networks of mineral molecules linked by covalent bonds. Geopolymers contain several molecular units that are produced in the geopolymerization process. The most important ones include polysilicate (-Si-O-Si-O-), polysialate (-Si-O-Al-O-), poly (sialate-siloxo) (-Si-O-Al-O-Si-O-), and poly (sialate-disiloxo) (-Si-O-Al-O- Si-O-Si-O-). The ferro-silico-aluminate (-Fe-O-Si-O-Al-O-) structure, which contains iron atoms, is much less known. Another criterion for the classification of geopolymers is the origin of the pozzolanic aluminosilicate material, and thus geopolymers based on metakaolin and fly ash can be distinguished [7,8,9,10]. Geopolymer composites are characterized, among others, by high compressive strength, low shrinkage, excellent frost resistance, high-temperature resistance, and acid resistance [11,12,13,14]. Due to the above-mentioned properties, interest in the described materials is constantly growing.

A significant advantage of geopolymers is their eco-friendliness. It has been noticed that the production of geopolymers causes a level of CO_2_ that is several times lower when compared to the production of Portland cement. According to various estimates, the synthesis of geopolymers consumes two to three times less energy than Portland cement [15]. Geopolymer materials can be applied as building materials, thermal insulators, adsorbents, catalysts, and fillers [16,17,18]. When properly designed, they can be used for insulation and fire resistance due to their low thermal conductivity [19,20,21]. Kamseu et al. [22] used iron-rich laterite in the production of geopolymers, which were combined with silica from rice husk ash. The results of the research showed that new phases that bind the geopolymeric binder were developed. The authors of paper [23] confirmed that the addition of iron strengthened the geopolymeric binder and caused a good cohesion between different particles, and also increased the number of pores. Kaze et al. [24] showed that the Si/Al ratio also has a great influence on the properties of calcined iron-rich laterite-based geopolymers. Davidovits [25] focused in his research on iron-rich geopolymers, and, at the same time, broke the current trend of iron having a negative impact on the mechanical properties of materials. He proved that even very high amounts of hematite (at the level of 40% in a sample) resulted in a high compressive strength of a sample, which was within the range of 70–90 MPa after 28 days of seasoning at room temperature.

The authors of publication [26] showed the effects of the sodium hydroxide ratio, the amount of external heat, and the partial replacement of Portland cement on fly ash-based geopolymer concrete. The early compressive strength, density, absorption, and permeable voids were measured, and the microstructure of the fly ash-based geopolymer paste was observed and characterized. The activating solution was a combination of silica fume, sodium hydroxide, and water. Experiments showed that the application of external heat plays a major role with regard to compressive strength. The results also show that early and final compressive strength gains, in case of the absence of external heat, can be improved by using Portland cement as a partial replacement for fly ash.

The aforementioned studies [21,22,23,24,25], related to the addition of iron and its incorporation into geopolymeric structures, were developed in the last two years. Scientists highlighted the new nature of their work and the poorly understood properties of innovative geopolymer building materials. Iron probably embeds into the octahedral structure of geopolymers, and replaces silicon or aluminum in their structure. Innovative materials modified in this way require their properties to be examined in a very wide spectrum. Geopolymers containing iron atoms in their structure are poorly understood materials.

The authors of this research noticed a significant lack of information regarding the study of the influence of the addition of iron on the thermal properties of modified geopolymers. The observed shortcomings in the literature data prompted the authors to conduct studies related to the use of iron in order to analyze the thermal properties of modified building materials. The aim of the research was to compile and compare the effect of adding various amounts of iron on the thermal parameters of the obtained composites. Geopolymer materials, unlike concrete, are innovative and little-known materials. It is therefore important, due to the growing interest in insulating building materials, to examine them and to indicate their actual thermal advantages.

The article presents the results of the measurements of the thermal conductivity λ, specific heat *C_p_*, thermal diffusivity *a*, and bulk density *ρ_b_*_1_ of the obtained geopolymer composites, which are often considered in separate publications. Apart from adding different amounts of iron, the geopolymers were modified with a different amount of sand and fireclay, which is not common with regard to the subject literature. Moreover, extensive thermal tests of all the modified geopolymers were carried out for two different levels of their moisture content: after stabilization under hygrothermal conditions, and after additional drying.

## 2. Materials and Methods

### 2.1. Materials

A geopolymer composite, which consisted of a metakaolin-based aluminosilicate binder that was activated with potassium silicate, was used in the tests. The binder was produced by Baucis LK (České Lupkové Závody, Nové Strašecí, Czech Republic). Moreover, iron powder was also added (Selkat, Kraków, Poland) in order to change the thermal properties of the obtained products. The content of iron in the used powder was equal to 99%, and 0.01% of carbon, 0.09% of oxygen, 0.01% of sulfur, and 0.18% of manganese were also found in the geopolymer. The grain diameter of the iron dust was mainly (67.5%) within the range of 45–150 μm. The addition of iron in the samples ranged from 0.5% to 2.5% in relation to the weight of the metakaolin. Reference samples were also made without the addition of iron filings.

The samples were modified with the addition of technical sand (Sklopísek Stře-leč, Hrdoňovice, Czech Republic) and fireclay (České Lupkové Závody, Nové Strašecí, Czech Republic) in a ratio of 1:1 and 1:1.2 to the weight of the metakaolin. The used technical sand was characterized by an exceptionally high SiO_2_ content (99.4%) and a low Fe_2_O_3_ content (0.04%). The grain size ranged from 0.315 mm to 0.800 mm, with an average size of 0.570 mm. The bulk density of the sand was 2.65 g/cm^3^. The fireclay used in the research was sedimentary rock, the dominant component of which was the clay mineral kaolinite. In terms of chemical composition, clay minerals are aluminosilicates with varying degrees of contamination with metal oxides and organic substances. The heat resistance that is characteristic for this ceramic material is due to the high Al_2_O_3_ content and, at the same time, the low content of melting admixtures. By firing these high-quality raw materials, thermally stable materials can be obtained. After their subsequent crushing, a material with high fire-resistance is obtained, which is used in the production of bricks and refractory concrete. The fireclay used in the tests, with a bulk density of 2.48 g/cm^3^, contained: 57.8% of SiO_2_, 36.4% of Al_2_O_3_, 2.6% of Fe_2_O_3_, and 3.2% of other metal oxides.

**Figure 1:**
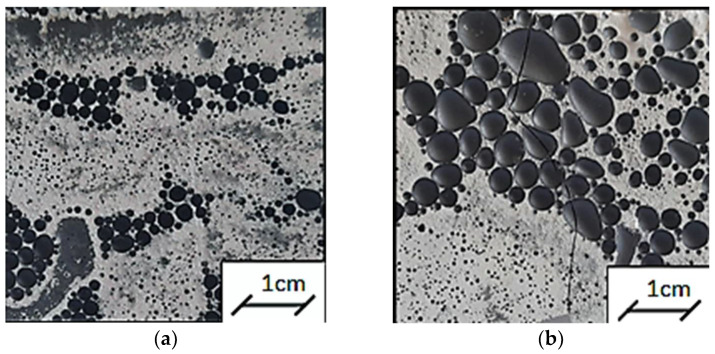
Reference samples used in the thermal tests after 28 days of conditioning under hygrothermal conditions: (**a**) RSS1, (**b**) RSF1.

**Figure 2:**
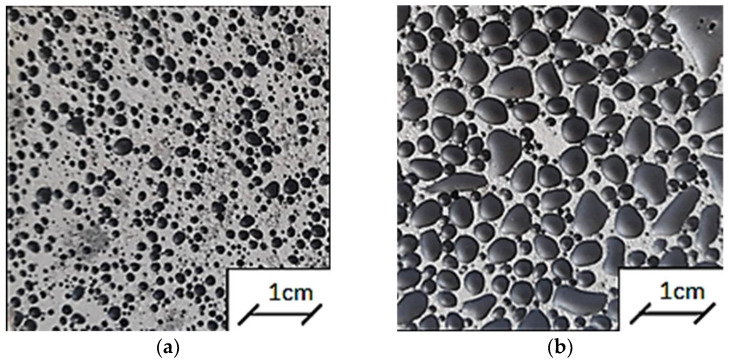
Reference samples used in the thermal tests after 28 days of conditioning under hygrothermal conditions: (**a**) RSS1.2, (**b**) RSF1.2.

**Figure 3:**
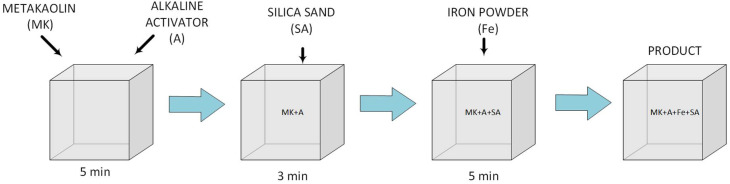
The preparation process of the modified geopolymers.

An activator was added to the aluminosilicate powder in a weight ratio of 1:0.9. The exact compositions of the geopolymeric slurries are summarized in Table 1. In order to activate the glassy phase of the geopolymer paste, the components were mixed for 5 min at room temperature in order to obtain a homogeneous mass of the specimen. Sand or fireclay was then added and mixed for another 3 min. At the final stage of preparing the product, iron powder was added and everything was stirred for another 5 min. The obtained geopolymer was placed in molds with dimensions of 10 cm × 10 cm × 10 cm and left for two hours. After this time, the samples and the mold were wrapped in foil for 48 h, and they were then removed from the molds and re-wrapped in foil in order to minimize water evaporation from the sample and to extend the geopolymer polymerization reaction time. The samples prepared in this way were left for a period of 26 days at a temperature of 20–22 °C and humidity of 52–54%. At a later stage, they were removed from the foil and seasoned for three days under the same temperature and humidity conditions, and further dried for 48 h in an incubator at 60 °C. Figure 1 and Figure 2 show photos of the studied geopolymer reference samples with different contents of sand and fireclay after the period of their conditioning under hygrothermal conditions. Both the increase in the amount of sand and fireclay caused an increase in the size of the pores of the tested samples. Six samples were made for each measurement series. The experimental procedure reported in Figure 3 shows how the raw materials were mixed in order to prepare all the modified geopolymers and the reference samples.

### 2.2. Methods

**Figure 4:**
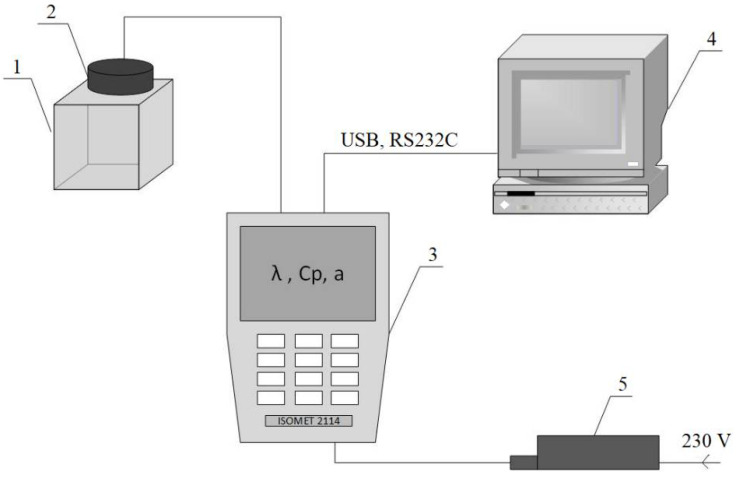
Scheme of the experimental stand for measuring the thermal properties of the building materials [30,31]: 1—tested sample, 2—probe, 3—Isomet 2114 device, 4—computer, 5—power supply.

The thermal parameters (thermal conductivity *λ*, volumetric heat capacity *C_v_*, and thermal diffusivity *a*) of the obtained samples were measured in two measuring cycles (before and after the drying process) using the Isomet 2114 device. The measurement method is based on conducting measurements under nonstationary conditions. Measurement methods that are based on undetermined heat conduction, in most cases, come down to the determination of thermal diffusivity, which is based on the measurement of temperature changes during a sample’s heating or cooling. Measurements that do not require a determined heat flow can be performed using the proposed measuring stand (Figure 4). The device analyzes temperature changes that result from the responses of the tested material to the flow of thermal pulses. These changes are measured by interchangeable probes that are connected with a gauge, which is, in turn, attached to a computer that records the results. During the measurement, the amount of heat generated by the device is known, and heat propagates radially through the sample. The increase in a sample’s temperature varies linearly with the logarithm of time. This relationship enables the thermal conductivity of the tested material to be directly obtained [27,28,29].

**Figure 5:**
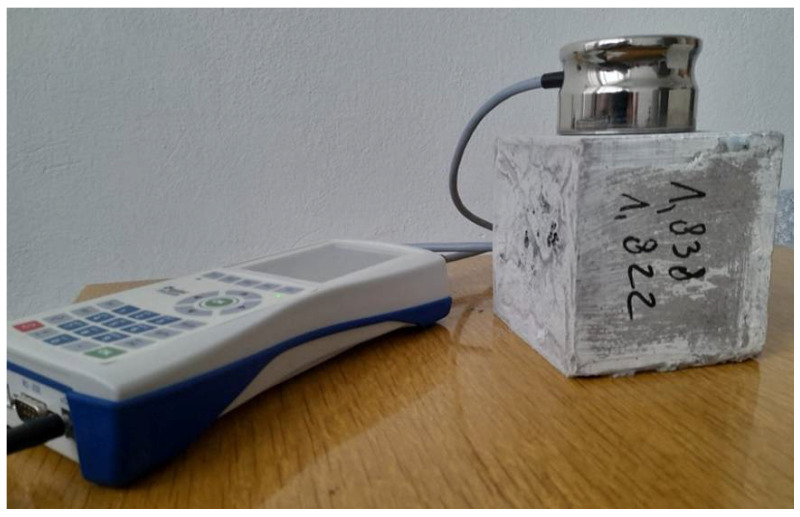
Investigation of the thermal properties of the modified geopolymer samples.

The device has a wide measuring range and can be used, among others, for insulating and building materials, plastics, glass, and minerals. The measuring range depends on the used probe and covers *λ* values from 0.015 to 6.0 W/(m·K), and *C_v_* values from 0.04 to 3 MJ/(m^3^·K). The measurement accuracy for the above ranges of thermal conductivity and volume specific heat is 5%. The meter has two optional types of probes: needle probes for soft materials, and surface probes for hard materials. Measurement data can be saved in the internal memory of the device, or can be exported to a computer. In the presented experiment, measurements were made using a surface probe (Figure 5).

## 3. Results and Discussion

### 3.1. Density Results

All the samples were weighed twice before and after the drying process. By knowing their dimensions and mass, the volumetric density of the samples *ρ_b_*_1_ was calculated from the simple Equation (1):

(1) $$\rho_{b1}=\frac{m}{V}$$

where *m* is the total mass of the specimen (with pores).

Additionally, the measured thermal parameters enabled the density *ρ_b_*_2_ to be calculated from dependence (2):

(2) $$\rho_{b2}=\frac{\lambda }{a\cdot C_{p}}$$

The density values *ρ_b_*_1_ and *ρ_b_*_2_ of the studied geopolymers, before and after drying, are summarized in Table 2.

**Figure 6:**
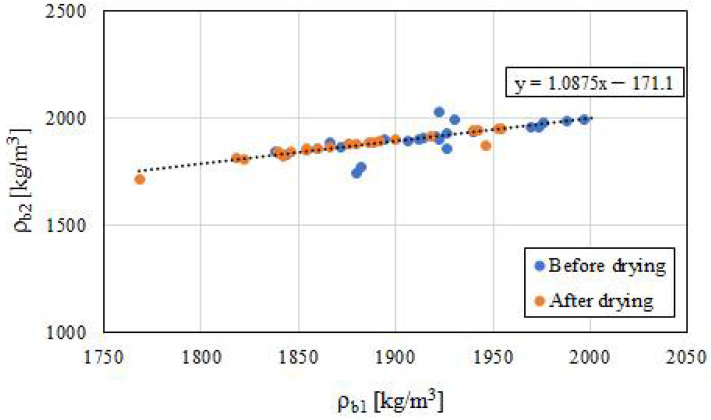
Graph of the dependence between *ρ_b_*_1_ and *ρ_b_*_2_ of all the tested samples, regardless of the seasoning process.

A high compatibility of the calculated density values obtained using both methods was noticed. However, better compatibilities were obtained in the case of the samples after drying. Due to a certain amount of water, the wet samples had less accurate measurement results. An obvious dependence can be seen—the samples after the drying process were characterized by a lower density than the samples seasoned under air-dry conditions. A generalized relationship *ρ_b_*_1_ = *f*(*ρ_b_*_2_) was proposed (Figure 6). Moreover, there was no clear correlation found between the amount of used iron powder and the obtained density of the composite—both before and after the drying process (Table 2).

### 3.2. Results of Thermal Properties and Their Discussion

Afterward, the thermal measurements of all the modified samples were performed, with the following being measured (always in six measurement series): thermal conductivity *λ*, volumetric heat capacity *C_v_,* and thermal diffusivity *a*. The results of the measurements, with the obtained statistical data, are presented in Tables S1–S8 in the Supplementary Materials. The tables include the results of the samples after being seasoned under hygro-thermal conditions and after their further drying in the dryer. Tables S1–S8 include, among others, the following statistical results: average value, standard deviation, coefficient of variation, outliers, critical values, and interquartile range. The specific heat *C_p_* expressed in units J/(kg·K) was obtained by dividing the measured volumetric heat capacity *C_v_* by the material volume density *ρ_b_*_1_. 

If the standard deviation of the random variable *X* is unknown, the distribution of the arithmetic average of sample $\bar{X\;}$ is very well approximated by the Student’s t-distribution. A detailed description of the assumptions of this distribution is included in [30]. It should be noted that if the tested random variable has the *N*(*µ, σ*) distribution and the standard deviation is not known, then the confidence interval is built using the Student’s *t*-distribution with the probability density expressed by Formula (3), where *Γ*(*x*) is the Euler gamma function:

(3) $$f\left(t,\;n\right)=\frac{\Gamma \left(\frac{n+1}{2}\right)}{\Gamma \left(\frac{n}{2}\right)\sqrt{n\pi }}{\left(1+\frac{t^{2}}{n}\right)}^{-\frac{n+1}{2}}$$

After the transformations, the following is obtained (4):

(4) $$P\left(\bar{X}-t_{n-1;\;\alpha /2}\frac{s}{\sqrt{n}}\leq \mu \leq \bar{X}+t_{n-1;\;\alpha /2}\frac{s}{\sqrt{n}}\right)=1-\alpha \;$$

where *α* is the assumed significance level, and 1 − *α* is the confidence level.

By having the results of *n* measurements, parameters such as the average $\bar{X\;}$ and standard deviation *s* were calculated for the sample. For all three measured values (thermal conductivity, specific heat, and thermal diffusivity), numerical intervals, within which the actual values can be found with a 95% probability, were estimated. For all the performed measurements, the measurement uncertainty was assessed based on the Student’s *t*-distribution. Based on statistical calculations, with the assumed confidence level of 95%, the confidence intervals of the measured thermal properties were determined. With a probability close to one, the sought values of the thermal parameters (*λ*, *C_v_, a*) of the modified geopolymers were within the ranges shown in Table 3.

**Figure 7:**
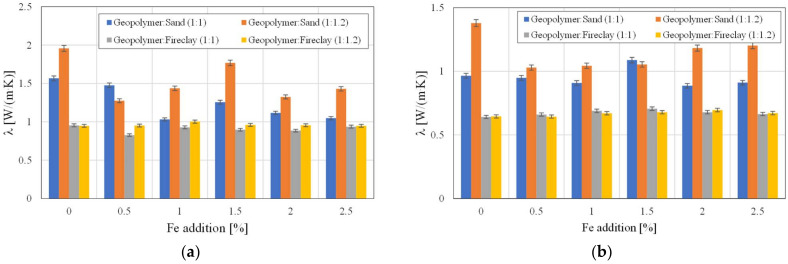
The values of thermal conductivity *λ* with regard to the used aggregates and the amount of added Fe: (**a**) before drying, (**b**) after drying.

All the samples before drying, due to the presence of water in their structure, had higher thermal conductivity values *λ*. Although the changes in the samples’ density *ρ_b_*_1_ before and after drying were not significant (Table 2), the values of thermal conductivity decreased by even 25–30% (Figure 7a,b, respectively). Such values of this parameter in the obtained composites (before drying) are due to small amounts of water, which has a high value of thermal conductivity. The samples with sand were characterized by higher values of thermal conductivity when compared to the samples with fireclay. All the samples containing the geopolymer with sand in the ratio of 1:1.2 had *λ* > 1 W/(m·K). The thermal conductivity value was 1.3791 W/(m·K) for the non-iron samples. For the samples with the addition of 2.5% Fe, the conductivity decreased to 1.2019 /(m·K). There was a decrease in this value by 12.8%. For the samples containing the geopolymer with sand in the ratio of 1:1.2, the thermal conductivity for the non-iron samples was 0.9657 W/(m·K) and 0.9109 W/(m·K) for the sample with the addition of 2.5% Fe. The thermal conductivity of the composites containing fireclay remained at the level of 0.67 W/(m·K) +/−5%. There were no such large changes in thermal conductivity, as was the case for the geopolymers with sand.

**Figure 8:**
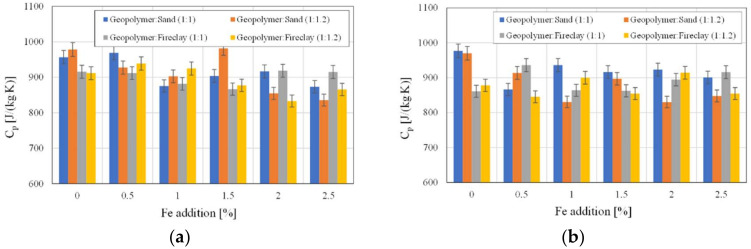
The values of specific heat *C_p_* with regard to the used aggregates and the amount of added Fe: (**a**) before drying, (**b**) after drying.

**Figure 9:**
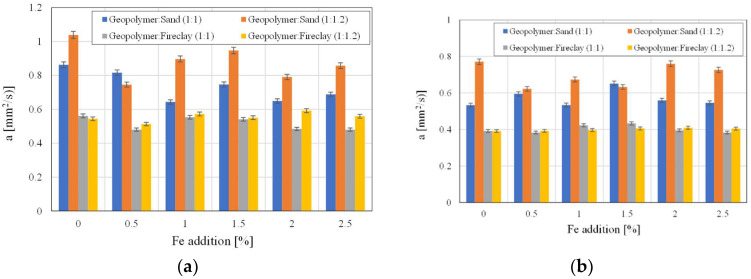
The thermal diffusivity values with regard to the used aggregates and the amount of added Fe: (**a**) before drying, (**b**) after drying.

The addition of fireclay caused an almost double decrease in the thermal conductivity of the composites when compared to the samples with the addition of sand. In the case of the samples with fireclay, no significant influence of the addition of iron on the values of thermal conductivity was noticed. However, in the case of using geopolymer with sand in the ratio of 1:1.2, it was noticed that with an increase in Fe, the thermal conductivity of the composite increased (Figure 7b). For the samples containing geopolymer with sand in the ratio of 1:1.2 and with a 2% addition of iron, the specific heat value was the lowest and amounted to 830 J/(kg·K). It was an almost 15% decrease in the specific heat value when compared to the samples without the addition of iron. A 2% error is marked on the bars in Figure 7, Figure 8 and Figure 9.

Figure 8a shows the values of the specific heat of the samples before the drying process, and Figure 8b shows those after the drying process. As a result of this process, the specific heat values decreased. The addition of iron in the samples containing sand after drying caused a decrease in the *C_p_* value. In the remaining samples, such a tendency was difficult to recognize. The results obtained for the samples containing fireclay were characterized by greater fluctuations, which is probably due to water being trapped in the pore structure (Figure 8b).

Figure 9a,b show the values of the thermal diffusivity of the samples before and after drying, respectively. The materials containing sand had higher values of diffusivity. The samples with the addition of fireclay had almost 50% lower values of this parameter when compared to the samples with sand. The samples with fireclay were characterized by a stable value of coefficient *a*, which did not depend on the amount of used iron. The materials with fireclay had a thermal diffusivity within the range from 0.3834 mm^2^/s to 0.4094 mm^2^/s. The samples containing sand had a thermal diffusivity of 0.5333–0.7706 mm^2^/s.

Thermal diffusivity can be defined as the ratio of the thermal conductivity of a substance to the heat storage capacity of this substance. At the beginning of the heat exchange process, materials with higher thermal diffusivity reach the determined state faster than composites with a lower value, which is due to the fact that they retain less thermal energy. Heat energy passes through such a material quickly, but once a determined state is reached, the heat flow rate will be the same. Materials with lower thermal diffusivity take longer to reach the determined state.

Fireclay is a ceramic material obtained by firing clay that is then ground. Fireclay products are characterized by high resistance to rapid temperature changes, which was noticed during the tests. After mixing with plastic clay, fireclay is used to make refractory materials. This feature is used in the construction of home tiled stoves, furnaces, and industrial stoves. Traditionally, fireclay mortar is a mixture of fireclay, raw clay, and additives in the form of Portland cement and sodium silicate. The use of fireclay in geopolymeric materials is a new and still little-known issue. The study of the unknown mechanical and thermal properties of the obtained innovative geopolymeric materials with the addition of fireclay should be continued. In addition, the reuse of fireclay bricks can generate significant benefits for the environmental sector. It also provides economic benefits for the building materials sector, which can use them as an alternative resource in the production of geopolymers.

The values of the thermal conductivity obtained in this study correspond very well with the results of research by other authors. Liu et al. [32] used oil palm shells and palm combustion ash in the production of geopolymers. The thermal conductivity of the material obtained in this way, amounting to 0.47 W/(m·K), was 22% and 48% lower when compared to conventional building materials such as concrete blocks and bricks, respectively. The authors of paper [33] used geopolymer fibers in various amounts as an additive to geopolymers. The test results showed that the modified geopolymeric materials changed their thermal conductivity within the range of 0.19–0.82 W/(m·K), and also that they can be successfully used as a thermal insulation material. In turn, paper [34] presents the production of a light, highly hydrophobic and perfectly thermally insulating geopolymer-aerogel composite. After being impregnated with silica aerogel, the composite had a nanoporous structure with a very low thermal conductivity value of 0.048 W/(m·K).

There are some publications describing the use of waste iron powder [35] and iron-rich slags [36] in the production of geopolymer products. These works, however, include tests related to the bending strength and compressive strength of the obtained composites. Due to the innovative nature of research concerning iron-containing geopolymers, there are not many studies on this subject, particularly with regard to the measurements of their thermal properties. A careful literature analysis revealed the publication of Nkwaju et al. [37], in which the authors presented the insulating properties of new geopolymer composites made of iron-rich laterite rocks with the addition of sugar cane waste and sodium silicate as a hardener. The authors showed that the thermal conductivity of iron-rich geopolymers, which were additionally modified with plant fibers, was equal to 0.55 W/(m·K). The samples without the waste content showed a thermal conductivity of 0.77 W/(m·K).

In the context of future research concerning innovative iron-containing geopolymeric materials, it is important to continuously investigate their thermal properties. This knowledge can be used to assess their suitability as insulating materials. At the same time, it is also important to establish the mechanical properties of such products. The authors of this publication are conducting such research that is yet to be published. In the authors’ opinion, only knowledge of both the thermal and mechanical parameters of new geopolymeric materials can contribute to providing a fuller picture of their potential usefulness in the construction industry.

## 4. Conclusions

- The addition of iron to the geopolymers caused significant changes in their thermal properties, such as thermal conductivity, specific heat, and thermal diffusivity.
- It was found that the addition of sand or fireclay had a significant influence on the thermal parameters of the obtained composites. The samples after the drying process were characterized by lower values of thermal parameters. The samples with sand were characterized by higher values of thermal conductivity when compared to the samples with fireclay. The highest values of thermal conductivity *λ*, exceeding 1 W(m·K), were exhibited by the samples containing the geopolymer with sand in the ratio of 1:1.2. The addition of fireclay caused a decrease in the thermal conductivity of the composites by at least 30% when compared to the samples with the addition of sand.
- The lowest value of the thermal conductivity coefficient *λ* was obtained for the geopolymer with metakaolin and fireclay. When the ratio of these components in the composite was 1:1, the value of thermal conductivity was 0.6413 W/(m·K), while in the case of the ratio of 1:1.2, it was equal to 0.6456 W/(m·K).
- The materials with sand had a thermal conductivity within the range from 0.8865 W/(m·K) to 1.3791 W/(m·K). The thermal conductivity value with the sand in the ratio of 1:1 was 1.3791 W/(m·K) for the non-iron samples. For the samples with the addition of 2.5% Fe, the conductivity decreased to 1.2019 /(m·K). There was a decrease in this value by 12.8%. The non-iron samples containing the geopolymer with sand in the ratio of 1:1.2 had a thermal conductivity equal to 0.9657 W/(m·K), and the samples containing the geopolymer with sand in the ratio of 1:1.2 with the addition of 2.5% Fe had a thermal conductivity equal to 0.9109 W/(m·K). The addition of iron reduced the thermal conductivity by 5.7%.
- In the samples containing fireclay, there was no significant influence of the added iron on the values of thermal conductivity. In turn, in the case of the geopolymer with sand, this influence was noticeable. It was most visible in the samples containing metakaolin and sand in the ratio of 1:1.2. Therefore, it was noticed that the thermal conductivity of the composites increased with the increase in the addition of Fe.
- In the case of specific heat and thermal diffusivity, the samples with sand were also characterized by higher values of these parameters when compared to the samples with fireclay. The samples with fireclay showed very good stability, i.e., there were no changes in thermal diffusivity with regard to the addition of iron.
- For the samples containing geopolymer with sand in the ratio of 1:1.2 and with a 2% addition of iron, the specific heat value was the lowest and amounted to 830 J/(kg·K). It was an almost 15% decrease in the specific heat value when compared to the samples without the addition of iron. The materials with fireclay had a thermal diffusivity within the range from 0.3834 mm^2^/s to 0.4094 mm^2^/s. The samples containing sand had a thermal diffusivity of 0.5333–0.7706 mm^2^/s.
- The calculated densities *ρ_b_*_1_ and *ρ_b_*_2_ of the obtained geopolymer composites did not differ significantly, despite the use of different calculation methods. In the first case, the density was calculated on the basis of the known weight and volume of the samples. In the second case, the density calculations were based on the known thermal properties of the samples.

## Figures and Tables

**Table 1:** Composition of the geopolymer pastes used in the research.

Tested Sample	Metakaolin [g]	Activator [g]	Sand [g]	Fireclay [g]	Fe Powder [g]
Reference sample (RSS1)	100	90	100	-	0
Geopolymer: Sand (1:1) + Fe0.5% (GS1Fe0.5)	100	90	100	-	0.5
Geopolymer: Sand (1:1) + Fe1.0% (GS1Fe1.0)	100	90	100	-	1.0
Geopolymer: Sand (1:1) + Fe1.5% (GS1Fe1.5)	100	90	100	-	1.5
Geopolymer: Sand (1:1) + Fe2.0% (GS1Fe2.0)	100	90	100	-	2.0
Geopolymer: Sand (1:1) + Fe2.5% (GS1Fe2.5)	100	90	100	-	2.5
Reference sample (RSS1.2)	100	90	120	-	0
Geopolymer: Sand (1:1.2) + Fe0.5% (GS1.2Fe0.5)	100	90	120	-	0.5
Geopolymer: Sand (1:1.2) + Fe1.0% (GS1.2Fe1.0)	100	90	120	-	1.0
Geopolymer: Sand (1:1.2) + Fe1.5% (GS1.2Fe1.5)	100	90	120	-	1.5
Geopolymer: Sand (1:1.2) + Fe2.0% (GS1.2Fe2.0)	100	90	120	-	2.0
Geopolymer: Sand (1:1.2) + Fe2.5% (GS1.2Fe2.0)	100	90	120	-	2.5
Reference sample (RSF1)	100	90	-	100	0
Geopolymer: Fireclay (1:1) + Fe0.5% (GF1Fe0.5)	100	90	-	100	0.5
Geopolymer: Fireclay (1:1) + Fe1.0% (GF1Fe1.0)	100	90	-	100	1.0
Geopolymer: Fireclay (1:1) + Fe1.5% (GF1Fe1.5)	100	90	-	100	1.5
Geopolymer: Fireclay (1:1) + Fe2.0% (GF1Fe2.0)	100	90	-	100	2.0
Geopolymer: Fireclay (1:1) + Fe2.5% (GF1Fe2.5)	100	90	-	100	2.5
Reference sample (RSF1.2)	100	90	-	120	0
Geopolymer: Fireclay (1:1.2) + Fe0.5% (GF1.2Fe0.5)	100	90	-	120	0.5
Geopolymer: Fireclay (1:1.2) + Fe1.0% (GF1.2Fe1.0)	100	90	-	120	1.0
Geopolymer: Fireclay (1:1.2) + Fe1.5% (GF1.2Fe1.5)	100	90	-	120	1.5
Geopolymer: Fireclay (1:1.2) + Fe2.0% (GF1.2Fe2.0)	100	90	-	120	2.0
Geopolymer: Fireclay (1:1.2) + Fe2.5% (GF1.2Fe2.5)	100	90	-	120	2.5

**Table 2:** The calculated bulk density *ρ_b_*_1_ and *ρ_b_*_2_ of the tested samples.

Tested Sample	Before Drying		After Drying	
	*ρ_b_*_1_ [kg/m^3^]	*ρ_b_*_2_ [kg/m^3^]	*ρ_b_*_1_ [kg/m^3^]	*ρ_b_*_2_ [kg/m^3^]
RSS1	1894	1899	1854	1854
GS1Fe0.5	1872	1868	1840	1841
GS1Fe1.0	1844	1831	1818	1818
GS1Fe1.5	1860	1861	1842	1824
GS1Fe2.0	1876	1876	1768	1716
GS1Fe2.5	1880	1743	1854	1854
RSS1.2	1926	1926	1846	1845
GS1.2Fe0.5	1838	1845	1822	1811
GS1.2Fe1.0	1882	1775	1866	1866
GS1.2Fe1.5	1922	1903	1860	1859
GS1.2Fe2.0	1974	1959	1946	1875
GS1.2Fe2.5	1997	1996	1953	1953
RSF1	1926	1857	1900	1900
GF1Fe0.5	1866	1888	1840	1840
GF1Fe1.0	1912	1904	1886	1886
GF1Fe1.5	1921	1913	1889	1890
GF1Fe2.0	1930	1990	1918	1918
GF1Fe2.5	1922	2132	1892	1892
RSF1.2	1914	1911	1880	1880
GF1.2Fe0.5	1976	1976	1942	1942
GF1.2Fe1.0	1906	1894	1876	1876
GF1.2Fe1.5	1988	1988	1954	1954
GF1.2Fe2.0	1940	1937	1854	1859
GF1.2Fe2.5	1970	1959	1940	1940

**Table 3:** Calculated confidence intervals of the measured thermal properties of the modified geopolymeric materials.

Tested Sample	Designated Confidence Intervals	
	Samples Conditioned in Hygrothermal Conditions	Samples Conditioned in the Dryer
RSS1	P (1.3787 ≤ *λ* ≤ 1.7545) = 1 − α	P (0.9622 ≤ *λ* ≤ 0.9691) = 1 − α
	P (914.0 ≤ *C_p_* ≤ 999.4) = 1 − α	P (976.0 ≤ *C_p_* ≤ 977.4) = 1 − α
	P (0.7951 ≤ *a* ≤ 0.9298) = 1 − α	P (0.5307 ≤ *a* ≤ 0.5359) = 1 − α
GS1Fe0.5	P (1.4302 ≤ *λ* ≤ 1.5226) = 1 − α	P (0.9416 ≤ *λ* ≤ 0.9559) = 1 − α
	P (934.4 ≤ *C_p_* ≤ 1003.1) = 1 − α	P (866.3 ≤ *C_p_* ≤ 866.9) = 1 − α
	P (0.8091 ≤ *a* ≤ 0.8229) = 1 − α	P (0.5902 ≤ *a* ≤ 0.5997) = 1 − α
GS1Fe1.0	P (0.9957 ≤ *λ* ≤ 1.0664) = 1 − α	P (0.9056 ≤ *λ* ≤ 0.9116) = 1 − α
	P (817.6 ≤ *C_p_* ≤ 933.4) = 1 − α	P (935.2 ≤ *C_p_* ≤ 936.9) = 1 − α
	P (0.6193 ≤ *a* ≤ 0.6672) = 1 − α	P (0.5326 ≤ *a* ≤ 0.5353) = 1 − α
GS1Fe1.5	P (1.1346 ≤ *λ* ≤ 1.3772) = 1 − α	P (1.0864 ≤ *λ* ≤ 1.0896) = 1 − α
	P (885.7 ≤ *C_p_* ≤ 922.0) = 1 − α	P (901.9 ≤ *C_p_* ≤ 930.8) = 1 − α
	P (0.6844 ≤ *a* ≤ 0.8086) = 1 − α	P (0.6489 ≤ *a* ≤ 0.6533) = 1 − α
GS1Fe2.0	P (0.9975 ≤ *λ* ≤ 1.2343) = 1 − α	P (0.8840 ≤ *λ* ≤ 0.8889) = 1 − α
	P (915.4 ≤ *C_p_* ≤ 917.8) = 1 − α	P (921.9 ≤ *C_p_* ≤ 924.7) = 1 − α
	P (0.5806 ≤ *a* ≤ 0.7171) = 1 − α	P (0.5167 ≤ *a* ≤ 0.6023) = 1 − α
GS1Fe2.5	P (1.0430 ≤ *λ* ≤ 1.0505) = 1 − α	P (0.9096 ≤ *λ* ≤ 0.9122) = 1 − α
	P (868.6 ≤ *C_p_* ≤ 878.1) = 1 − α	P (899.6 ≤ *C_p_* ≤ 902.4) = 1 − α
	P (0.5585 ≤ *a* ≤ 0.8166) = 1 − α	P (0.5438 ≤ *a* ≤ 0.5466) = 1 − α
RSS1.2	P (1.9437 ≤ *λ* ≤ 1.9710) = 1 − α	P (1.3765 ≤ *λ* ≤ 1.3817) = 1 − α
	P (969.4 ≤ *C_p_* ≤ 987.3) = 1 − α	P (969.3 ≤ *C_p_* ≤ 970.7) = 1 − α
	P (1.0350 ≤ *a* ≤ 1.0425) = 1 − α	P (0.7684 ≤ *a* ≤ 0.7727) = 1 − α
GS1.2Fe0.5	P (1.0935 ≤ *λ* ≤ 1.4564) = 1 − α	P (1.0202 ≤ *λ* ≤ 1.0389) = 1 − α
	P (870.6 ≤ *C_p_* ≤ 984.0) = 1 − α	P (910.8 ≤ *C_p_* ≤ 916.6) = 1 − α
	P (0.6843 ≤ *a* ≤ 0.8062) = 1 − α	P (0.6150 ≤ *a* ≤ 0.6296) = 1 − α
GS1.2Fe1.0	P (1.4347 ≤ *λ* ≤ 1.4384) = 1 − α	P (0.9854 ≤ *λ* ≤ 1.1019) = 1 − α
	P (870.9 ≤ *C_p_* ≤ 934.3) = 1 − α	P (829.9 ≤ *C_p_* ≤ 831.6) = 1 − α
	P (0.8947 ≤ *a* ≤ 0.8982) = 1 − α	P (0.6386 ≤ *a* ≤ 0.7081) = 1 − α
GS1.2Fe1.5	P (1.7000 ≤ *λ* ≤ 1.8362) = 1 − α	P (1.0523 ≤ *λ* ≤ 1.0560) = 1 − α
	P (896.1 ≤ *C_p_* ≤ 1066.9) = 1 − α	P (895.6 ≤ *C_p_* ≤ 898.2) = 1 − α
	P (0.8620 ≤ *a* ≤ 1.0311) = 1 − α	P (0.6305 ≤ *a* ≤ 0.6339) = 1 − α
GS1.2Fe2.0	P (1.2145 ≤ *λ* ≤ 1.4333) = 1 − α	P (1.1701 ≤ *λ* ≤ 1.1952) = 1 − α
	P (797.8 ≤ *C_p_* ≤ 911.4) = 1 − α	P (828.3 ≤ *C_p_* ≤ 832.3) = 1 − α
	P (0.6733 ≤ *a* ≤ 0.9080) = 1 − α	P (0.7148 ≤ *a* ≤ 0.8045) = 1 − α
GS1.2Fe2.5	P (1.3518 ≤ *λ* ≤ 1.5082) = 1 − α	P (1.2001 ≤ *λ* ≤ 1.2037) = 1 − α
	P (825.8 ≤ *C_p_* ≤ 845.3) = 1 − α	P (846.6 ≤ *C_p_* ≤ 849.3) = 1 − α
	P (0.8007 ≤ *a* ≤ 0.9141) = 1 − α	P (0.7234 ≤ *a* ≤ 0.7285) = 1 − α
RSF1	P (0.8500 ≤ *λ* ≤ 1.0576) = 1 − α	P (0.6374 ≤ *λ* ≤ 0.6451) = 1 − α
	P (887.8 ≤ *C_p_* ≤ 943.2) = 1 − α	P (860.6 ≤ *C_p_* ≤ 861.4) = 1 − α
	P (0.5500 ≤ *a* ≤ 0.5717) = 1 − α	P (0.3891 ≤ *a* ≤ 0.3950) = 1 − α
GF1Fe0.5	P (0.8099 ≤ *λ* ≤ 0.8442) = 1 − α	P (0.6593 ≤ *λ* ≤ 0.6619) = 1 − α
	P (909.4 ≤ *C_p_* ≤ 914.0) = 1 − α	P (935.4 ≤ *C_p_* ≤ 936.4) = 1 − α
	P (0.4573 ≤ *a* ≤ 0.5035) = 1 − α	P (0.3819 ≤ *a* ≤ 0.3853) = 1 − α
GF1Fe1.0	P (0.9089 ≤ *λ* ≤ 0.9483) = 1 − α	P (0.6884 ≤ *λ* ≤ 0.6904) = 1 − α
	P (824.9 ≤ *C_p_* ≤ 937.8) = 1 − α	P (863.5 ≤ *C_p_* ≤ 864.4) = 1 − α
	P (0.5062 ≤ *a* ≤ 0.6005) = 1 − α	P (0.4223 ≤ *a* ≤ 0.4239) = 1 − α
GF1Fe1.5	P (0.7955 ≤ *λ* ≤ 0.9988) = 1 − α	P (0.7021 ≤ *λ* ≤ 0.7100) = 1 − α
	P (834.3 ≤ *C_p_* ≤ 898.8) = 1 − α	P (862.1 ≤ *C_p_* ≤ 863.0) = 1 − α
	P (0.4605 ≤ *a* ≤ 0.6218) = 1 − α	P (0.4306 ≤ *a* ≤ 0.4356) = 1 − α
GF1Fe2.0	P (0.8748 ≤ *λ* ≤ 0.8954) = 1 − α	P (0.6781 ≤ *λ* ≤ 0.8791) = 1 − α
	P (902.2 ≤ *C_p_* ≤ 934.6) = 1 − α	P (892.4 ≤ *C_p_* ≤ 896.7) = 1 − α
	P (0.4447 ≤ *a* ≤ 0.5240) = 1 − α	P (0.3930 ≤ *a* ≤ 0.3980) = 1 − α
GF1Fe2.5	P (0.9129 ≤ *λ* ≤ 0.9616) = 1 − α	P (0.6633 ≤ *λ* ≤ 0.6655) =1 − α
	P (884.3 ≤ *C_p_* ≤ 945.7) = 1 − α	P (915.2 ≤ *C_p_* ≤ 916.8) = 1 − α
	P (0.4548 ≤ *a* ≤ 0.5061) = 1 − α	P (0.3826 ≤ *a* ≤ 0.3841) = 1 − α
RSF1.2	P (0.9425 ≤ *λ* ≤ 0.9534) = 1 − α	P (0.6427 ≤ *λ* ≤ 0.6485) = 1 − α
	P (870.7 ≤ *C_p_* ≤ 952.5) = 1 − α	P (876.0 ≤ *C_p_* ≤ 880.2) = 1 − α
	P (0.5198 ≤ *a* ≤ 0.5684) = 1 − α	P (0.3888 ≤ *a* ≤ 0.3935) = 1 − α
GF1.2Fe0.5	P (0.9468 ≤ *λ* ≤ 0.9556) = 1 − α	P (0.6435 ≤ *λ* ≤ 0.6448) = 1 − α
	P (922.6 ≤ *C_p_* ≤ 954.8) = 1 − α	P (844.3 ≤ *C_p_* ≤ 846.4) = 1 − α
	P (0.5048 ≤ *a* ≤ 0.5210) = 1 − α	P (0.3916 ≤ *a* ≤ 3932) = 1 − α
GF1.2Fe1.0	P (0.9991 ≤ *λ* ≤ 1.0046) = 1 − α	P (0.6673 ≤ *λ* ≤ 0.6728) = 1 − α
	P (839.7 ≤ *C_p_* ≤ 1009.5) = 1 − α	P (898.8 ≤ *C_p_* ≤ 900.7) = 1 − α
	P (0.5195 ≤ *a* ≤ 0.6248) = 1 − α	P (0.3935 ≤ *a* ≤ 0.4003) = 1 − α
GF1.2Fe1.5	P (0.9561 ≤ *λ* ≤ 0.9639) = 1 − α	P (0.6775 ≤ *λ* ≤ 0.6793) = 1 − α
	P (867.4 ≤ *C_p_* ≤ 887.0) = 1 − α	P (854.3 ≤ *C_p_* ≤ 855.2) = 1 − α
	P (0.5461 ≤ *a* ≤ 0.5551) = 1 − α	P (0.4052 ≤ *a* ≤ 0.4072) = 1 − α
GF1.2Fe2.0	P (0.8385 ≤ *λ* ≤ 1.0716) = 1 − α	P (0.6924 ≤ *λ* ≤ 0.6992) = 1 − α
	P (807.4 ≤ *C_p_* ≤ 858.4) = 1 − α	P (909.7 ≤ *C_p_* ≤ 918.9) = 1 − α
	P (0.5102 ≤ *a* ≤ 0.6738) = 1 − α	P (0.4048 ≤ *a* ≤ 0.4139) = 1 − α
GF1.2Fe2.5	P (0.9380 ≤ *λ* ≤ 0.9588) = 1 − α	P (0.6691 ≤ *λ* ≤ 0.6740) = 1 − α
	P (786.7 ≤ *C_p_* ≤ 944.2) = 1 − α	P (852.0 ≤ *C_p_* ≤ 857.5) = 1 − α
	P (0.5145 ≤ *a* ≤ 0.6041) = 1 − α	P (0.4017 ≤ *a* ≤ 4083) = 1 − α

## Data Availability

The data presented in this study are available on request from the corresponding author.

## Supplementary Materials

The following supporting information can be downloaded at: www.mdpi.com/article/10.3390/polym14102009/s1, Table S1. The obtained values of the thermal parameters of the geopolymer samples with sand (1:1), and the calculated statistical parameters after 28 days of conditioning in 20–22 °C and a relative humidity of 52–54%. Table S2. The obtained values of the thermal parameters of the geopolymer samples with sand (1:1.2), and the calculated statistical parameters after 28 days of conditioning in 20–22 °C and a relative humidity of 52–54%. Table S3. The obtained values of the thermal parameters of the geopolymer samples with fireclay (1:1), and the calculated statistical parameters after 28 days of conditioning in 20–22 °C and a relative humidity of 52–54%. Table S4. The obtained values of the thermal parameters of the geopolymer samples with fireclay (1:1.2), and the calculated statistical parameters after 28 days of conditioning in 20–22°C and a relative humidity of 52–54%. Table S5. The obtained values of the thermal parameters of the geopolymer samples with sand (1:1), and the calculated statistical parameters after 3 days of conditioning in 60 °C. Table S6. The obtained values of the thermal parameters of the geopolymer samples with sand (1:1.2), and the calculated statistical parameters after 3 days of conditioning in 60°C. Table S7. The obtained values of the thermal parameters of the geopolymer samples with fireclay (1:1), and the calculated statistical parameters after 3 days of conditioning in 60 °C. Table S8. The obtained values of the thermal parameters of the geopolymer samples with fireclay (1:1.2), and the calculated statistical parameters after 3 days of conditioning in 60°C.
