# Supplementary material for: Determination of the Thermal Parameters of Geopolymers Modified with Iron Powder
Source: Polymers (Basel). 2022 May 13;14(10):2009. doi: 10.3390/polym14102009 (PMC9148025; doi:10.3390/polym14102009)
Supplement: Supplementary file 1 [file polymers-14-02009-s001.zip › polymers-1717953-supplementary.pdf]

polymers

MDPI

Supplementary

Table S1. The obtained values of the thermal parameters of the geopolymer samples with sand (1:1), and the calculated statistical parameters after 28 days of conditioning in 20-22°C and a relative humidity of 52-54%

Statistical parameters	Thermal properties of sample RSS1			
	λ[W/(m·K)]	C_v [MJ/(m³·K)]	C_p [J/(kg·K)]	a [mm²/s]
Quartile, *Q1*	1.3726	1.7418	919.6	0.7881
Median, *M*	1.6039	1.8102	955.8	0.8841
Quartile, *Q3*	1.7264	1.8825	993.9	0.9153
Interquartile range, *IQR = (Q3 - Q1)*	0.3538	0.1407	74.3	0.1272
Higher outlier, *HO = Q3 + 1.5·IQR*	2.2571	2.0936	1105.4	1.1061
Lower outlier, *LO = Q1 - 1.5·IQR*	0.8419	1.5308	808.2	0.5973
Average value, $\bar{X}$	1.5666	1.8120	956.7	0.8625
Standard deviation, *s*	0.1790	0.0771	40.7	0.0642
Coefficient of variation, *CV* [%]	11.43	4.25	4.25	7.44
Upper critical value, *UCV*	1.7545	1.8929	999.4	0.9298
Lower critical value, *LCV*	1.3787	1.7311	914.0	0.7951
Statistical parameters	Thermal properties of sample GS1Fe0.5			
	λ[W/(m·K)]	C_v [MJ/(m³·K)]	C_p [J/(kg·K)]	a [mm²/s]
Quartile, *Q1*	1.4383	1.7589	939.6	0.8138
Median, *M*	1.4742	1.8146	969.3	0.8157
Quartile, *Q3*	1.5180	1.8675	997.6	0.8218
Interquartile range, *IQR = (Q3 - Q1)*	0.0797	0.1086	58.0	0.0080
Higher outlier, *HO = Q3 + 1.5·IQR*	1.6376	2.0304	1084.6	0.8338
Lower outlier, *LO = Q1 - 1.5·IQR*	1.3188	1.5960	852.6	0.8018
Average value, $\bar{X}$	1.4764	1.8135	968.8	0.8160
Standard deviation, *s*	0.0440	0.0613	32.7	0.0066
Coefficient of variation, *CV* [%]	2.98	3.38	3.38	0.81
Upper critical value, *UCV*	1.5226	1.8778	1003.1	0.8229
Lower critical value, *LCV*	1.4302	1.7492	934.4	0.8091
Statistical parameters	Thermal properties of sample GS1Fe1.0			
	λ[W/(m·K)]	C_v [MJ/(m³·K)]	C_p [J/(kg·K)]	a [mm²/s]
Quartile, *Q1*	1.0000	1.5068	817.1	0.6222
Median, *M*	1.0311	1.6300	883.9	0.6436
Quartile, *Q3*	1.0618	1.7053	924.8	0.6640
Interquartile range, *IQR = (Q3 - Q1)*	0.0618	0.1985	107.7	0.0418
Higher outlier, *HO = Q3 + 1.5·IQR*	1.1545	2.0031	1086.3	0.7267
Lower outlier, *LO = Q1 - 1.5·IQR*	0.9073	1.2091	655.7	0.5595
Average value, $\bar{X}$	1.0311	1.6145	875.5	0.6432
Standard deviation, *s*	0.0337	0.1017	55.2	0.0228
Coefficient of variation, *CV* [%]	3.27	6.30	6.30	3.54
Upper critical value, *UCV*	1.0664	1.7212	933.4	0.6672
Lower critical value, *LCV*	0.9957	1.5077	817.6	0.6193
Statistical parameters	Thermal properties of sample GS1Fe1.5			
	λ [W/(m·K)]	C_v [MJ/(m³·K)]	C_p [J/(kg·K)]	a [mm²/s]
Quartile, *Q1*	1.1470	1.6591	891.9	0.6920

Median, *M*	1.2550	1.6631	894.1	0.7422
Quartile, *Q3*	1.3584	1.7153	922.2	0.7944
Interquartile range, *IQR = (Q3 - Q1)*	0.2114	0.0562	30.3	0.1024
Higher outlier, *HO = Q3 + 1.5·IQR*	1.6755	1.7996	967.5	0.9480
Lower outlier, *LO = Q1 - 1.5·IQR*	0.8299	1.5748	846.7	0.5384
Average value, $\bar{X}$	1.2559	1.6811	903.8	0.7465
Standard deviation, *s*	0.1156	0.0322	17.3	0.0591
Coefficient of variation, *CV* [%]	9.20	1.92	1.92	7.92
Upper critical value, *UCV*	1.3772	1.7149	922.0	0.8086
Lower critical value, *LCV*	1.1346	1.6473	885.7	0.6844

Statistical parameters	Thermal properties of sample GS1Fe2.0			
	λ[W/(m·K)]	C_v [MJ/(m^3·K)]	C_p [J/(kg·K)]	a [mm^2/s]
Quartile, *Q1*	1.0129	1.7173	915.4	0.5895
Median, *M*	1.1153	1.7196	916.6	0.6481
Quartile, *Q3*	1.2175	1.7210	917.4	0.7078
Interquartile range, *IQR = (Q3 - Q1)*	0.2046	0.0037	2.0	0.1183
Higher outlier, *HO = Q3 + 1.5·IQR*	1.5244	1.7266	920.3	0.8853
Lower outlier, *LO = Q1 - 1.5·IQR*	0.7060	1.7118	912.4	0.4121
Average value, $\bar{X}$	1.1159	1.7195	916.6	0.6488
Standard deviation, *s*	0.1128	0.0022	1.1	0.0650
Coefficient of variation, *CV* [%]	10.11	0.13	0.13	10.02
Upper critical value, *UCV*	1.2343	1.7217	917.8	0.7171
Lower critical value, *LCV*	0.9975	1.7172	915.4	0.5806

Statistical parameters	Thermal properties of sample GS1Fe2.5			
	λ[W/(m·K)]	C_v [MJ/(m^3·K)]	C_p [J/(kg·K)]	a [mm^2/s]
Quartile, *Q1*	1.0483	1.6348	869.6	0.6361
Median, *M*	1.0480	1.6420	873.4	0.6371
Quartile, *Q3*	1.0495	1.6490	877.1	0.6407
Interquartile range, *IQR = (Q3 - Q1)*	0.0012	0.0142	7.5	0.0046
Higher outlier, *HO = Q3 + 1.5·IQR*	1.0584	1.6703	888.4	0.6476
Lower outlier, *LO = Q1 - 1.5·IQR*	1.0348	1.6135	858.2	0.6292
Average value, $\bar{X}$	1.0468	1.6419	873.4	0.6875
Standard deviation, *s*	0.0036	0.0085	4.5	0.1230
Coefficient of variation, *CV* [%]	0.34	0.52	0.52	17.88
Upper critical value, *UCV*	1.0505	1.6509	878.1	0.8166
Lower critical value, *LCV*	1.0430	1.6330	868.6	0.5585

Table S2. The obtained values of the thermal parameters of the geopolymer samples with sand (1:1.2), and the calculated statistical parameters after 28 days of conditioning in 20-22°C and a relative humidity of 52-54%

Statistical parameters	Thermal properties of sample RSS1.2			
	λ [W/(m·K)]	C_v [MJ/(m^3·K)]	C_p [J/(kg·K)]	a [mm^2/s]
Quartile, *Q1*	1.9462	1.8685	970.1	1.0361
Median, *M*	1.9545	1.8840	978.2	1.0396
Quartile, *Q3*	1.9718	1.8983	985.6	1.0416
Interquartile range, *IQR = (Q3 - Q1)*	0.0256	0.0298	15.5	0.0055
Higher outlier, *HO = Q3 + 1.5·IQR*	2.0102	1.9430	1008.8	1.0499
Lower outlier, *LO = Q1 - 1.5·IQR*	1.9078	1.8238	946.9	1.0279
Average value, $\bar{X}$	1.9573	1.8843	978.3	1.0388
Standard deviation, *s*	0.0130	0.0164	8.5	0.0036
Coefficient of variation, *CV* [%]	0.66	0.87	0.87	0.34

Upper critical value, *UCV*	1.9710	1.9015	987.3	1.0425
Lower critical value, *LCV*	1.9437	1.8671	969.4	1.0350

Statistical parameters	Thermal properties of sample GS1.2Fe0.5			
	λ [W/(m·K)]	C_v [MJ/(m³·K)]	C_p [J/(kg·K)]	a [mm²/s]
Quartile, *Q1*	1.1167	1.6136	877.9	0.6920
Median, *M*	1.2758	1.0731	926.6	0.7448
Quartile, *Q3*	1.4297	1.7939	976.0	0.7978
Interquartile range, *IQR = (Q3 - Q1)*	0.3130	0.1803	98.1	0.1058
Higher outlier, *HO = Q3 + 1.5·IQR*	1.8992	2.0644	1123.2	0.9565
Lower outlier, *LO = Q1 - 1.5·IQR*	0.6472	1.3432	730.8	0.5333
Average value, $\bar{X}$	1.2750	1.7044	927.3	0.7452
Standard deviation, *s*	0.1729	0.0993	54.0	0.0581
Coefficient of variation, *CV* [%]	13.56	5.83	5.83	7.79
Upper critical value, *UCV*	1.4564	1.8086	984.0	0.8062
Lower critical value, *LCV*	1.0935	1.6002	870.6	0.6843

Statistical parameters	Thermal properties of sample GS1.2Fe1.0			
	λ [W/(m·K)]	C_v [MJ/(m³·K)]	C_p [J/(kg·K)]	a [mm²/s]
Quartile, *Q1*	1.4332	1.6489	876.1	0.8950
Median, *M*	1.4367	1.6922	899.1	0.8960
Quartile, *Q3*	1.4370	1.7351	921.9	0.8977
Interquartile range, *IQR = (Q3 - Q1)*	0.0038	0.0862	45.8	0.0027
Higher outlier, *HO = Q3 + 1.5·IQR*	1.4427	1.8644	990.6	0.9018
Lower outlier, *LO = Q1 - 1.5·IQR*	1.4275	1.5196	807.4	0.8910
Average value, $\bar{X}$	1.4365	1.6987	902.6	0.8965
Standard deviation, *s*	0.0018	0.0568	30.2	0.0016
Coefficient of variation, *CV* [%]	0.12	3.34	3.34	0.18
Upper critical value, *UCV*	1.4384	1.7583	934.3	0.8982
Lower critical value, *LCV*	1.4347	1.6391	870.9	0.8947

Statistical parameters	Thermal properties of sample GS1.2Fe1.5			
	λ [W/(m·K)]	C_v [MJ/(m³·K)]	C_p [J/(kg·K)]	a [mm²/s]
Quartile, *Q1*	1.8236	1.7206	895.2	0.9214
Median, *M*	1.8304	1.9809	1030.6	0.9519
Quartile, *Q3*	1.9002	1.9861	1033.4	0.9854
Interquartile range, *IQR = (Q3 - Q1)*	0.0766	0,2655	138.2	0.0640
Higher outlier, *HO = Q3 + 1.5·IQR*	2.0151	2.3844	1240.6	1.0814
Lower outlier, *LO = Q1 - 1.5·IQR*	1.7087	1.3224	688.0	0.8254
Average value, $\bar{X}$	1.7681	1.8864	981.5	0.9466
Standard deviation, *s*	0.0601	0.1564	81.4	0.0746
Coefficient of variation, *CV* [%]	3.40	8.29	8.29	7.88
Upper critical value, *UCV*	1.8362	2.0505	1066.9	1.0311
Lower critical value, *LCV*	1.7000	1.7223	896.1	0.8620

Statistical parameters	Thermal properties of sample GS1.2Fe2.0			
	λ [W/(m·K)]	C_v [MJ/(m³·K)]	C_p [J/(kg·K)]	a [mm²/s]
Quartile, *Q1*	1.2291	1.5890	805.0	0.6879
Median, *M*	1.3223	1.6869	854.5	0.7903
Quartile, *Q3*	1.4197	1.7851	904.3	0.8932
Interquartile range, *IQR = (Q3 - Q1)*	0.1906	0.1961	99.3	0.2053
Higher outlier, *HO = Q3 + 1.5·IQR*	1.7056	2.0793	1053.3	1.2012
Lower outlier, *LO = Q1 - 1.5·IQR*	0.9432	1.2949	656.0	0.3800

Average value, $\bar{X}$	1.3239	1.6871	854.6	0.7906
Standard deviation, *s*	0.1043	0.1068	54.1	0.1119
Coefficient of variation, *CV* [%]	7.87	6.33	6.33	14.15
Upper critical value, *UCV*	1.4333	1.7992	911.4	0.9080
Lower critical value, *LCV*	1.2145	1.5750	797.8	0.6733

Statistical parameters	Thermal properties of sample GS1.2Fe2.5			
	λ [W/(m·K)]	C_v [MJ/(m³·K)]	C_p [J/(kg·K)]	*a* [mm²/s]
Quartile, *Q1*	1.3620	1.6516	827.0	0.8070
Median, *M*	1.4290	1.6682	835.4	0.8573
Quartile, *Q3*	1.4999	1.6852	843.9	0.9076
Interquartile range, *IQR = (Q3 - Q1)*	0.1379	0.0336	16.9	0.1006
Higher outlier, *HO = Q3 + 1.5·IQR*	1.7068	1.7356	869.1	1.0585
Lower outlier, *LO = Q1 - 1.5·IQR*	1.1552	1.6012	801.8	0.6561
Average value, $\bar{X}$	1.4300	1.6686	835.6	0.8574
Standard deviation, *s*	0.0745	1.6686	9.3	0.0540
Coefficient of variation, *CV* [%]	5.21	1.11	1.11	6.30
Upper critical value, *UCV*	1.5082	1.6881	845.3	0.9141
Lower critical value, *LCV*	1.3518	1.6491	825.8	0.8007

Table S3. The obtained values of the thermal parameters of the geopolymer samples with fireclay (1:1), and the calculated statistical parameters after 28 days of conditioning in 20-22°C and a relative humidity of 52-54%

Statistical parameters	Thermal properties of sample RSF1			
	λ [W/(m·K)]	C_v [MJ/(m³·K)]	C_p [J/(kg·K)]	*a* [mm²/s]
Quartile, *Q1*	0.9754	1.7171	891.5	0.5506
Median, *M*	0.9898	1.7643	916.0	0.5607
Quartile, *Q3*	0.9974	1.8110	940.3	0.5689
Interquartile range, *IQR = (Q3 - Q1)*	0.0220	0.0939	48.8	0.0183
Higher outlier, *HO = Q3 + 1.5·IQR*	1.9519	1.9519	1013.4	0.5964
Lower outlier, *LO = Q1 - 1.5·IQR*	0.9424	1.5763	818.4	0.5232
Average value, $\bar{X}$	0.9538	1.7633	915.5	0.5609
Standard deviation, *s*	0.0916	0.0508	26.4	0.0103
Coefficient of variation, *CV* [%]	9.60	2.88	2.88	1.84
Upper critical value, *UCV*	1.0576	1.8166	943.2	0.5717
Lower critical value, *LCV*	0.8500	1.7100	887.8	0.5500

Statistical parameters	Thermal properties of sample GF1Fe0.5			
	λ [W/(m·K)]	C_v [MJ/(m³·K)]	C_p [J/(kg·K)]	*a* [mm²/s]
Quartile, *Q1*	0.7887	1.6879	904.6	0.4651
Median, *M*	0.8202	1.6931	907.3	0.4866
Quartile, *Q3*	0.8659	1.7160	919.6	0.5035
Interquartile range, *IQR = (Q3 - Q1)*	0.0772	0.0281	15.0	0.0384
Higher outlier, *HO = Q3 + 1.5·IQR*	0.9817	1.7582	942.2	0.5611
Lower outlier, *LO = Q1 - 1.5·IQR*	0.6729	1.6458	882.0	0.4075
Average value, $\bar{X}$	0.8270	1.7013	911.7	0.4804
Standard deviation, *s*	0.0182	0.0045	2.4	0.0245
Coefficient of variation, *CV* [%]	2.20	0.27	0.27	5.11
Upper critical value, *UCV*	0.8442	1.7055	914.0	0.5035
Lower critical value, *LCV*	0.8099	1.6970	909.4	0.4573

Statistical parameters	Thermal properties of sample GF1Fe1.0			
	λ [W/(m·K)]	C_v [MJ/(m³·K)]	C_p [J/(kg·K)]	*a* [mm²/s]

Quartile, *Q1*	0.9112	1.5917	832.5	0.5122
Median, *M*	0.9286	1.6856	881.6	0.5529
Quartile, *Q3*	0.9457	1.7792	930.5	0.5943
Interquartile range, *IQR = (Q3 - Q1)*	0.0345	0.1875	98.0	0.0821
Higher outlier, *HO = Q3 + 1.5·IQR*	0.9975	2.0605	1077.6	0.7175
Lower outlier, *LO = Q1 - 1.5·IQR*	0.8595	1.3105	685.4	0.3891
Average value, $\bar{X}$	0.9286	1.6851	881.3	0.5533
Standard deviation, *s*	0.0188	0.1028	53.8	0.0449
Coefficient of variation, *CV* [%]	2.02	2.57	2.57	8.12
Upper critical value, *UCV*	0.9483	1.7931	937.8	0.6005
Lower critical value, *LCV*	0.9089	1.5772	824.9	0.5062

Statistical parameters	Thermal properties of sample GF1Fe1.5			
	λ [W/(m·K)]	C_v [MJ/(m^3·K)]	C_p [J/(kg·K)]	*a* [mm^2/s]
Quartile, *Q1*	0.8010	1.6111	838.7	0.4696
Median, *M*	0.9047	1.6598	864.0	0.5424
Quartile, *Q3*	0.9857	1.7102	890.3	0.6118
Interquartile range, *IQR = (Q3 - Q1)*	0.1847	0.0991	51.6	0.1422
Higher outlier, *HO = Q3 + 1.5·IQR*	1.2628	1.8589	967.6	0.8251
Lower outlier, *LO = Q1 - 1.5·IQR*	0.5240	1.4625	761.3	0.2563
Average value, $\bar{X}$	0.8972	1.6647	866.6	0.5412
Standard deviation, *s*	0.0969	0.0590	30.7	0.0768
Coefficient of variation, *CV* [%]	10.80	3.55	3.55	14.20
Upper critical value, *UCV*	0.9988	1.7266	898.8	0.6218
Lower critical value, *LCV*	0.7955	1.6027	834.3	0.4605

Statistical parameters	Thermal properties of sample GF1Fe2.0			
	λ [W/(m·K)]	C_v [MJ/(m^3·K)]	C_p [J/(kg·K)]	*a* [mm^2/s]
Quartile, *Q1*	0.8769	1.7455	904.4	0.4963
Median, *M*	0.8858	1.7727	918.5	0.4984
Quartile, *Q3*	0.8938	1.7996	932.4	0.5021
Interquartile range, *IQR = (Q3 - Q1)*	0.0169	0.0541	28.0	0.0058
Higher outlier, *HO = Q3 + 1.5·IQR*	0.9192	1.8808	974.5	0.5108
Lower outlier, *LO = Q1 - 1.5·IQR*	0.8516	1.6644	862.4	0.4876
Average value, $\bar{X}$	0.8851	1.7726	918.4	0.4844
Standard deviation, *s*	0.0099	0.0298	15.4	0.0378
Coefficient of variation, *CV* [%]	1.12	1.68	1.68	7.81
Upper critical value, *UCV*	0.8954	1.8038	934.6	0.5240
Lower critical value, *LCV*	0.8747	1.7413	902.2	0.4447

Statistical parameters	Thermal properties of sample GF1Fe2.5			
	λ [W/(m·K)]	C_v [MJ/(m^3·K)]	C_p [J/(kg·K)]	*a* [mm^2/s]
Quartile, *Q1*	0.9175	1.7269	898.5	0.4651
Median, *M*	0.9259	1.7337	902.0	0.4866
Quartile, *Q3*	0.9744	1.8697	972.8	0.5035
Interquartile range, *IQR = (Q3 - Q1)*	0.0569	0.1428	74.3	0.0384
Higher outlier, *HO = Q3 + 1.5·IQR*	1.0598	2.0839	1084.2	0.5611
Lower outlier, *LO = Q1 - 1.5·IQR*	0.8322	1.5127	787.0	0.4075
Average value, $\bar{X}$	0.9372	1.7586	915.0	0.4804
Standard deviation, *s*	0.0259	0.0627	32.6	0.0273
Coefficient of variation, *CV* [%]	2.76	3.56	3.56	5.67
Upper critical value, *UCV*	0.9616	1.8176	945.7	0.5061
Lower critical value, *LCV*	0.9129	1.6996	884.3	0.4548

Table S4. The obtained values of the thermal parameters of the geopolymer samples with fireclay (1:1.2), and the calculated statistical parameters after 28 days of conditioning in 20-22°C and a relative humidity of 52-54%

Statistical parameters	Thermal properties of sample RSF1.2			
	λ [W/(m·K)]	C_v [MJ/(m³·K)]	C_p [J/(kg·K)]	a [mm²/s]
Quartile, *Q1*	0.9431	1.6776	876.5	0.5230
Median, *M*	0.9477	1.7441	911.2	0.5429
Quartile, *Q3*	0.9497	1.8134	947.4	0.5632
Interquartile range, *IQR = (Q3 - Q1)*	0.0066	0.1358	70.9	0.0402
Higher outlier, *HO = Q3 + 1.5·IQR*	0.9596	2.0171	1053.9	0.6235
Lower outlier, *LO = Q1 - 1.5·IQR*	0.9332	1.4739	770.1	0.4627
Average value, $\bar{X}$	0.9479	1.7448	911.6	0.5441
Standard deviation, *s*	0.0052	0.0745	38.9	0.0232
Coefficient of variation, *CV* [%]	0.55	4.27	4.27	4.26
Upper critical value, *UCV*	0.9534	1.8230	952.5	0.5684
Lower critical value, *LCV*	0.9425	1.6666	870.7	0.5198
Statistical parameters	Thermal properties of sample GF1.2Fe0.5			
	λ [W/(m·K)]	C_v [MJ/(m³·K)]	C_p [J/(kg·K)]	a [mm²/s]
Quartile, *Q1*	0.9490	1.8280	925.1	0.5054
Median, *M*	0.9516	1.8541	938.3	0.5120
Quartile, *Q3*	0.9534	1.8839	953.4	0.5213
Interquartile range, *IQR = (Q3 - Q1)*	0.0044	0.0559	28.3	0.0159
Higher outlier, *HO = Q3 + 1.5·IQR*	0.9600	1.9678	995.8	0.5453
Lower outlier, *LO = Q1 - 1.5·IQR*	0.9424	1.7442	882.7	0.4816
Average value, $\bar{X}$	0.9512	1.8549	938.7	0.5129
Standard deviation, *s*	0.0042	0.0303	15.3	0.0077
Coefficient of variation, *CV* [%]	0.44	1.63	1.63	1.51
Upper critical value, *UCV*	0.9556	1.8866	954.8	0.5210
Lower critical value, *LCV*	0.9468	1.8231	922.6	0.5048
Statistical parameters	Thermal properties of sample GF1.2Fe1.0			
	λ [W/(m·K)]	C_v [MJ/(m³·K)]	C_p [J/(kg·K)]	a [mm²/s]
Quartile, *Q1*	1.0003	1.6214	850.7	0.5259
Median, *M*	1.0007	1.7623	924.6	0.5725
Quartile, *Q3*	1.0050	1.9026	998.2	0.6174
Interquartile range, *IQR = (Q3 - Q1)*	0.0047	0.2812	147,5	0.0915
Higher outlier, *HO = Q3 + 1.5·IQR*	1.0121	2.3244	1219.5	0.7547
Lower outlier, *LO = Q1 - 1.5·IQR*	0.9933	1.1996	629.4	0.3887
Average value, $\bar{X}$	1.0019	1.7623	924.6	0.5722
Standard deviation, *s*	0.0026	0.1542	80.9	0.0502
Coefficient of variation, *CV* [%]	0.26	8.75	8.75	8.77
Upper critical value, *UCV*	1.0046	1.9242	1009.5	0.6248
Lower critical value, *LCV*	0.9991	1.6005	839.7	0.5195
Statistical parameters	Thermal properties of sample GF1.2Fe1.5			
	λ [W/(m·K)]	C_v [MJ/(m³·K)]	C_p [J/(kg·K)]	a [mm²/s]
Quartile, *Q1*	0.9569	1.7284	869.4	0.5477
Median, *M*	0.9596	1.7389	874.7	0.5515
Quartile, *Q3*	0.9607	1.7651	887.9	0.5542
Interquartile range, *IQR = (Q3 - Q1)*	0.0038	0.0367	18.5	0.0065
Higher outlier, *HO = Q3 + 1.5·IQR*	0.9664	1.8202	915.6	0.5640
Lower outlier, *LO = Q1 - 1.5·IQR*	0.9512	1.6734	841.7	0.5380

Average value, $\bar{X}$	0.9600	1.7438	877.2	0.5506
Standard deviation, *s*	0.0037	0.0186	9.3	0.0043
Coefficient of variation, *CV* [%]	0.38	1.06	1.06	0.78
Upper critical value, *UCV*	0.9639	1.7633	887.0	0.5551
Lower critical value, *LCV*	0.9561	1.7243	867.4	0.5461

Statistical parameters	Thermal properties of sample GF1.2Fe2.0			
	λ [W/(m·K)]	C_v [MJ/(m^3·K)]	C_p [J/(kg·K)]	*a* [mm^2/s]
Quartile, *Q1*	0.9117	1.5709	809.7	0.5513
Median, *M*	0.9139	1.6158	832.9	0.5667
Quartile, *Q3*	0.9159	1.6614	856.4	0.5818
Interquartile range, *IQR = (Q3 - Q1)*	0.0042	0.0905	46.7	0.0305
Higher outlier, *HO = Q3 + 1.5·IQR*	0.9222	1.7972	926.4	0.6276
Lower outlier, *LO = Q1 - 1.5·IQR*	0.9054	1.4352	739.8	0.5056
Average value, $\bar{X}$	0.9551	1.6158	832.9	0.5920
Standard deviation, *s*	0.1028	0.0471	24.3	0.0722
Coefficient of variation, *CV* [%]	10.77	2.91	2.91	12.19
Upper critical value, *UCV*	1.0716	1.6653	858.4	0.6738
Lower critical value, *LCV*	0.8385	1.5664	807.4	0.5102

Statistical parameters	Thermal properties of sample GF1.2Fe2.5			
	λ [W/(m·K)]	C_v [MJ/(m^3·K)]	C_p [J/(kg·K)]	*a* [mm^2/s]
Quartile, *Q1*	0.9391	1.5699	796.9	0.5203
Median, *M*	0.9480	1.7039	864.9	0.5598
Quartile, *Q3*	0.9579	1.8409	934.5	0.5980
Interquartile range, *IQR = (Q3 - Q1)*	0.0188	0.2710	137.6	0.0777
Higher outlier, *HO = Q3 + 1.5·IQR*	0.9861	2.2474	1140.8	0.7146
Lower outlier, *LO = Q1 - 1.5·IQR*	0.9109	1.1634	590.6	0.4038
Average value, $\bar{X}$	0.9484	1.7049	865.4	0.5593
Standard deviation, *s*	0.0099	0.1478	75.0	0.0427
Coefficient of variation, *CV* [%]	1.04	8.67	8.67	7.64
Upper critical value, *UCV*	0.9588	1.8600	944.2	0.6041
Lower critical value, *LCV*	0.9380	1.5498	786.7	0.5145

Table S5. The obtained values of the thermal parameters of the geopolymer samples with sand (1:1), and the calculated statistical parameters after 3 days of conditioning in 60°C

Statistical parameters	Thermal properties of sample RSS1			
	λ[W/(m·K)]	C_v [MJ/(m^3·K)]	C_p [J/(kg·K)]	*a* [mm^2/s]
Quartile, *Q1*	0.9621	1.8099	976.2	0.5313
Median, *M*	0.9628	1.8106	976.6	0.5323
Quartile, *Q3*	0.9638	1.8118	977.2	0.5362
Interquartile range, *IQR = (Q3 - Q1)*	0.0017	0.0019	1.0	0.0049
Higher outlier, *HO = Q3 + 1.5·IQR*	0.9655	1.8137	978.3	0.5411
Lower outlier, *LO = Q1 - 1.5·IQR*	0.9604	1.8080	975.2	0.5264
Average value, $\bar{X}$	0.9657	1.8108	976.7	0.5333
Standard deviation, *s*	0.0033	0.0013	0.7	0.0025
Coefficient of variation, *CV* [%]	0.35%	0.07	0.07	0.47
Upper critical value, *UCV*	0.9691	1.8122	977.4	0.5359
Lower critical value, *LCV*	0.9622	1.8094	976.0	0.5307

Statistical parameters	Thermal properties of sample GS1Fe0.5			
	λ[W/(m·K)]	C_v [MJ/(m^3·K)]	C_p [J/(kg·K)]	*a* [mm^2/s]

Quartile, *Q1*	0.9401	1.5940	866.2	0.5895
Median, *M*	0.9455	1.5941	866.3	0.5964
Quartile, *Q3*	0.9508	1.5945	866.4	0.5990
Interquartile range, *IQR = (Q3 - Q1)*	0.0107	0.0005	0.2	0.0095
Higher outlier, *HO = Q3 + 1.5·IQR*	0.9615	1.5950	866.6	0.6085
Lower outlier, *LO = Q1 - 1.5·IQR*	0.9294	1.5935	866.0	0.5800
Average value, $\bar{X}$	0.9487	1.5942	866.4	0.5949
Standard deviation, *s*	0.0068	0.0004	0.1	0.0045
Coefficient of variation, *CV* [%]	0.72	0.02	0.02	0.76
Upper critical value, *UCV*	0.9559	1.5946	866.6	0.5997
Lower critical value, *LCV*	0.9416	1.5938	866.3	0.5902

Statistical parameters	Thermal properties of sample GS1Fe1.0			
	λ[W/(m·K)]	C_v [MJ/(m^3·K)]	C_p [J/(kg·K)]	*a* [mm^2/s]
Quartile, *Q1*	0.9050	1.7006	935.4	0.5325
Median, *M*	0.9070	1.7007	935.5	0.5344
Quartile, *Q3*	0.9089	1.7038	935.5	0.5351
Interquartile range, *IQR = (Q3 - Q1)*	0.0039	0.0032	0.1	0.0026
Higher outlier, *HO = Q3 + 1.5·IQR*	0.9128	1.7070	935.6	0.5377
Lower outlier, *LO = Q1 - 1.5·IQR*	0.9011	1.6974	935.3	0.5299
Average value, $\bar{X}$	0.9086	1.7018	936.1	0.5340
Standard deviation, *s*	0.0029	0.0017	0.8	0.0013
Coefficient of variation, *CV* [%]	0.32	0.10	0.08	0.25
Upper critical value, *UCV*	0.9116	1.7036	936.9	0.5353
Lower critical value, *LCV*	0.9056	1.6999	935.2	0.5326

Statistical parameters	Thermal properties of sample GS1Fe1.5			
	λ [W/(m·K)]	C_v [MJ/(m^3·K)]	C_p [J/(kg·K)]	*a* [mm^2/s]
Quartile, *Q1*	1.0861	1.6699	906.6	0.6497
Median, *M*	1.0869	1.6738	908.7	0.6505
Quartile, *Q3*	1.0877	1.7202	933.9	0.6531
Interquartile range, *IQR = (Q3 - Q1)*	0.0016	0.0503	27.3	0.0034
Higher outlier, *HO = Q3 + 1.5·IQR*	1.0893	1.7705	961.2	0.6565
Lower outlier, *LO = Q1 - 1.5·IQR*	1.0845	1.6196	879.3	0.6463
Average value, $\bar{X}$	1.0880	1.6879	916.3	0.6511
Standard deviation, *s*	0.0015	0.0254	13.8	0.0021
Coefficient of variation, *CV* [%]	0.14	1.50	1.50	0.32
Upper critical value, *UCV*	1.0896	1.7145	930.8	0.6533
Lower critical value, *LCV*	1.0864	1.6613	901.9	0.6489

Statistical parameters	Thermal properties of sample GS1Fe2.0			
	λ[W/(m·K)]	C_v [MJ/(m^3·K)]	C_p [J/(kg·K)]	*a* [mm^2/s]
Quartile, *Q1*	0.8835	1.6299	921.9	0.5424
Median, *M*	0.8857	1.6325	923.4	0.5429
Quartile, *Q3*	0.8879	1.6335	923.9	0.5438
Interquartile range, *IQR = (Q3 - Q1)*	0.0044	0.0036	2.0	0.0014
Higher outlier, *HO = Q3 + 1.5·IQR*	0.8923	1.6371	926.0	0.5452
Lower outlier, *LO = Q1 - 1.5·IQR*	0.8791	1.6263	919.9	0.5410
Average value, $\bar{X}$	0.8865	1.6324	923.3	0.5595
Standard deviation, *s*	0.0023	0.0024	1.3	0.0408
Coefficient of variation, *CV* [%]	0.26	0.15	0.15	7.29
Upper critical value, *UCV*	0.8889	1.6349	924.7	0.6023
Lower critical value, *LCV*	0.8840	1.6299	921.9	0.5167

Statistical parameters	Thermal properties of sample GS1Fe2.5			
	λ[W/(m·K)]	C_v [MJ/(m^3·K)]	C_p [J/(kg·K)]	a [mm^2/s]
Quartile, *Q1*	0.9088	1.6689	900.2	0.5439
Median, *M*	0.9102	1.6694	900.4	0.5454
Quartile, *Q3*	0.9115	1.6730	902.4	0.5463
Interquartile range, *IQR = (Q3 - Q1)*	0.0027	0.0041	2.2	0.0024
Higher outlier, *HO = Q3 + 1.5·IQR*	0.9142	1.6771	904.6	0.5487
Lower outlier, *LO = Q1 - 1.5·IQR*	0.9061	1.6648	898.0	0.5415
Average value, $\bar{X}$	0.9109	1.6704	901.0	0.5452
Standard deviation, *s*	0.0013	0.0025	1.3	0.0013
Coefficient of variation, *CV* [%]	0.14	0.15	0.15	0.24
Upper critical value, *UCV*	0.9122	1.6730	902.4	0.5466
Lower critical value, *LCV*	0.9096	1.6678	899.6	0.5438

Table S6. The obtained values of the thermal parameters of the geopolymer samples with sand (1:1.2), and the calculated statistical parameters after 3 days of conditioning in 60°C

Statistical parameters	Thermal properties of sample RSS1.2			
	λ [W/(m·K)]	C_v [MJ/(m^3·K)]	C_p [J/(kg·K)]	a [mm^2/s]
Quartile, *Q1*	1.3755	1.7892	969.2	0.7684
Median, *M*	1.3783	1.7910	970.2	0.7713
Quartile, *Q3*	1.3805	1.7913	970.4	0.7718
Interquartile range, *IQR = (Q3 - Q1)*	0.0050	0.0021	1.1	0.0034
Higher outlier, *HO = Q3 + 1.5·IQR*	1.3855	1.7934	971.5	0.7752
Lower outlier, *LO = Q1 - 1.5·IQR*	1.3705	1.7871	968.1	0.7650
Average value, $\bar{X}$	1.3791	1.7906	970.0	0.7706
Standard deviation, *s*	0.0025	0.0013	0.7	0.0020
Coefficient of variation, *CV* [%]	0.18	0.07	0.07	0.27
Upper critical value, *UCV*	1.3817	1.7920	970.7	0.7727
Lower critical value, *LCV*	1.3765	1.7893	969.3	0.7684
**Statistical parameters**	**Thermal properties of sample GS1.2Fe0.5**			
	λ [W/(m·K)]	C_v [MJ/(m^3·K)]	C_p [J/(kg·K)]	a [mm^2/s]
Quartile, *Q1*	1.0215	1.6583	910.2	0.6139
Median, *M*	1.0230	1.6674	915.1	0.6248
Quartile, *Q3*	1.0245	1.6684	915.7	0.6253
Interquartile range, *IQR = (Q3 - Q1)*	0.0030	0.0101	5.5	0.0114
Higher outlier, *HO = Q3 + 1.5·IQR*	1.0275	1.6785	921.2	0.6367
Lower outlier, *LO = Q1 - 1.5·IQR*	1.0185	1.6482	904.6	0.6025
Average value, $\bar{X}$	1.0296	1.6647	913.7	0.6223
Standard deviation, *s*	0.0089	0.0051	2.8	0.0069
Coefficient of variation, *CV* [%]	0.87	0.31	0.31	1.11
Upper critical value, *UCV*	1.0389	1.6701	916.6	0.6296
Lower critical value, *LCV*	1.0202	1.6594	910.8	0.6150
**Statistical parameters**	**Thermal properties of sample GS1.2Fe1.0**			
	λ [W/(m·K)]	C_v [MJ/(m^3·K)]	C_p [J/(kg·K)]	a [mm^2/s]
Quartile, *Q1*	0.9759	1.5503	830.8	0.6307
Median, *M*	1.0264	1.5506	831.0	0.6944
Quartile, *Q3*	1.0769	1.5510	831.2	0.6951
Interquartile range, *IQR = (Q3 - Q1)*	0.1010	0.0007	0.4	0.0644
Higher outlier, *HO = Q3 + 1.5·IQR*	1.1779	1.5517	831.6	0.7595

Lower outlier, *LO = Q1 - 1.5·IQR*	0.8749	1.5496	830.4	0.5663
Average value, $\bar{X}$	1.0437	1.5502	830.7	0.6734
Standard deviation, *s*	0.0555	0.0015	0.8	0.0331
Coefficient of variation, *CV* [%]	5.32	0.10	0.10	4.92
Upper critical value, *UCV*	1.1019	1.5517	831.6	0.7081
Lower critical value, *LCV*	0.9854	1.5486	829.9	0.6386

Statistical parameters	Thermal properties of sample GS1.2Fe1.5			
	λ [W/(m·K)]	C_v [MJ/(m^3·K)]	C_p [J/(kg·K)]	a [mm^2/s]
Quartile, *Q1*	1.0521	1.6666	896.0	0.6311
Median, *M*	1.0528	1.6687	897.1	0.6313
Quartile, *Q3*	1.0548	1.6702	898.0	0.6341
Interquartile range, *IQR = (Q3 - Q1)*	0.0027	0.0036	1.9	0.0030
Higher outlier, *HO = Q3 + 1.5·IQR*	1.0575	1.6738	899.9	0.6371
Lower outlier, *LO = Q1 - 1.5·IQR*	1.0494	1.6630	894.1	0.6281
Average value, $\bar{X}$	1.0542	1.6683	896.9	0.6322
Standard deviation, *s*	0.0018	0.0023	1.2	0.0016
Coefficient of variation, *CV* [%]	0.17	0.14	0.14	0.25
Upper critical value, *UCV*	1.0560	1.6707	898.2	0.6339
Lower critical value, *LCV*	1.0523	1.6659	895.6	0.6305

Statistical parameters	Thermal properties of sample GS1.2Fe2.0			
	λ [W/(m·K)]	C_v [MJ/(m^3·K)]	C_p [J/(kg·K)]	a [mm^2/s]
Quartile, *Q1*	1.1608	1.6137	829.2	0.7384
Median, *M*	1.1802	1.6159	830.3	0.7269
Quartile, *Q3*	1.1889	1.6181	831.5	0.8139
Interquartile range, *IQR = (Q3 - Q1)*	0.0281	0.0044	2.3	0.0755
Higher outlier, *HO = Q3 + 1.5·IQR*	1.2170	1.6225	833.8	0.8894
Lower outlier, *LO = Q1 - 1.5·IQR*	1.1327	1.6093	827.0	0.6629
Average value, $\bar{X}$	1.1826	1.6158	830.3	0.7596
Standard deviation, *s*	0.0119	0.0037	1.9	0.0428
Coefficient of variation, *CV* [%]	1.01%	0.23%	0.23%	5.63%
Upper critical value, *UCV*	1.1952	1.6196	832.3	0.8045
Lower critical value, *LCV*	1.1701	1.6119	828.3	0.7148

Statistical parameters	Thermal properties of sample GS1.2Fe2.5			
	λ [W/(m·K)]	C_v [MJ/(m^3·K)]	C_p [J/(kg·K)]	a [mm^2/s]
Quartile, *Q1*	1.2000	1.6539	846.9	0.7237
Median, *M*	1.2005	1.6543	847.0	0.7257
Quartile, *Q3*	1.2012	1.6595	849.7	0.7285
Interquartile range, *IQR = (Q3 - Q1)*	0.0012	0.0056	2.9	0.0048
Higher outlier, *HO = Q3 + 1.5·IQR*	1.2024	1.6651	852.6	0.7333
Lower outlier, *LO = Q1 - 1.5·IQR*	1.1989	1.6483	844.0	0.7189
Average value, $\bar{X}$	1.2019	1.6558	847.8	0.7259
Standard deviation, *s*	0.0017	0.0030	1.5	0.0024
Coefficient of variation, *CV* [%]	0.14	0.18	0.18	0.33
Upper critical value, *UCV*	1.2037	1.6590	849.5	0.7285
Lower critical value, *LCV*	1.2001	1.6527	846.2	0.7234

Table S7. The obtained values of the thermal parameters of the geopolymer samples with fireclay (1:1), and the calculated statistical parameters after 3 days of conditioning in 60°C

Statistical parameters	Thermal properties of sample RSF1			
	λ [W/(m·K)]	C_v [MJ/(m³·K)]	C_p [J/(kg·K)]	a [mm²/s]
Quartile, *Q1*	0.6384	1.6352	860.5	0.3901
Median, *M*	0.6386	1.6357	860.7	0.3904
Quartile, *Q3*	0.6386	1.6370	860.9	0.3956
Interquartile range, *IQR = (Q3 - Q1)*	0.0002	0.0018	0.4	0.0055
Higher outlier, *HO = Q3 + 1.5·IQR*	0.6388	1.6388	861.3	0.4011
Lower outlier, *LO = Q1 - 1.5·IQR*	0.6382	1.6334	860.2	0.3846
Average value, $\bar{X}$	0.6413	1.6359	861.0	0.3920
Standard deviation, *s*	0.0037	0.0009	0.4	0.0028
Coefficient of variation, *CV* [%]	0.57	0.06	0.05	0.72
Upper critical value, *UCV*	0.6451	1.6369	861.4	0.3950
Lower critical value, *LCV*	0.6374	1.6350	860.6	0.3891
**Statistical parameters**	**Thermal properties of sample GF1Fe0.5**			
	λ [W/(m·K)]	C_v [MJ/(m³·K)]	C_p [J/(kg·K)]	a [mm²/s]
Quartile, *Q1*	0.6578	1.7212	935.2	0.3817
Median, *M*	0.6596	1.7217	935.5	0.3829
Quartile, *Q3*	0.6606	1.7232	935.7	0.3838
Interquartile range, *IQR = (Q3 - Q1)*	0.0028	0.0020	0.5	0.0021
Higher outlier, *HO = Q3 + 1.5·IQR*	0.6634	1.7252	936.2	0.3859
Lower outlier, *LO = Q1 - 1.5·IQR*	0.6550	1.7192	934.7	0.3796
Average value, $\bar{X}$	0.6606	1.7221	935.9	0.3836
Standard deviation, *s*	0.0012	0.0013	0.5	0.0016
Coefficient of variation, *CV* [%]	0.18	0.08	0.05	0.42
Upper critical value, *UCV*	0.6619	1.7235	936.5	0.3853
Lower critical value, *LCV*	0.6593	1.7207	935.4	0.3819
**Statistical parameters**	**Thermal properties of sample GF1Fe1.0**			
	λ [W/(m·K)]	C_v [MJ/(m³·K)]	C_p [J/(kg·K)]	a [mm²/s]
Quartile, *Q1*	0.6883	1.6288	863.2	0.4224
Median, *M*	0.6886	1.6295	863.8	0.4231
Quartile, *Q3*	0.6895	1.6300	864.0	0.4238
Interquartile range, *IQR = (Q3 - Q1)*	0.0012	0.0012	0.8	0.0014
Higher outlier, *HO = Q3 + 1.5·IQR*	0.6907	1.6312	864.8	0.4252
Lower outlier, *LO = Q1 - 1.5·IQR*	0.6871	1.6276	862.4	0.4210
Average value, $\bar{X}$	0.6894	1.6294	863.9	0.4231
Standard deviation, *s*	0.0009	0.0009	0.4	0.0008
Coefficient of variation, *CV* [%]	0.13	0.05	0.05	0.18
Upper critical value, *UCV*	0.6904	1.6303	864.4	0.4239
Lower critical value, *LCV*	0.6884	1.6285	863.5	0.4223
**Statistical parameters**	**Thermal properties of sample GF1Fe1.5**			
	λ [W/(m·K)]	C_v [MJ/(m³·K)]	C_p [J/(kg·K)]	a [mm²/s]
Quartile, *Q1*	0.7005	1.6288	862.3	0.4308
Median, *M*	0.7047	1.6295	862.6	0.4331
Quartile, *Q3*	0.7082	1.6300	862.9	0.4354
Interquartile range, *IQR = (Q3 - Q1)*	0.0077	0.0012	0.6	0.0046
Higher outlier, *HO = Q3 + 1.5·IQR*	0.7159	1.6312	863.5	0.4400
Lower outlier, *LO = Q1 - 1.5·IQR*	0.6928	1.6276	861.6	0.4262

Average value, $\bar{X}$	0.7060	1.6294	862.6	0.4331
Standard deviation, *s*	0.0038	0.0009	0.5	0.0024
Coefficient of variation, *CV* [%]	0.54	0.05	0.05	0.55
Upper critical value, *UCV*	0.7100	1.6303	863.0	0.4356
Lower critical value, *LCV*	0.7021	1.6285	862.1	0.4306

Statistical parameters	Thermal properties of sample GF1Fe2.0			
	λ [W/(m·K)]	C_v [MJ/(m^3·K)]	C_p [J/(kg·K)]	*a* [mm^2/s]
Quartile, *Q1*	0.6775	1.7121	892.6	0.3942
Median, *M*	0.6783	1.7156	894.5	0.3951
Quartile, *Q3*	0.6783	1.7195	896.5	0.3978
Interquartile range, *IQR = (Q3 - Q1)*	0.0008	0.0074	3.9	0.0036
Higher outlier, *HO = Q3 + 1.5·IQR*	0.6791	1.7269	900.4	0.4014
Lower outlier, *LO = Q1 - 1.5·IQR*	0.6767	1.7047	888.8	0.3906
Average value, $\bar{X}$	0.6786	1.7157	894.5	0.3955
Standard deviation, *s*	0.0005	0.0039	2.0	0.0024
Coefficient of variation, *CV* [%]	0.07	0.23	0.23	0.60
Upper critical value, *UCV*	0.6791	1.7198	896.7	0.3980
Lower critical value, *LCV*	0.6781	1.7116	892.4	0.3930

Statistical parameters	Thermal properties of sample GF1Fe2.5			
	λ [W/(m·K)]	C_v [MJ/(m^3·K)]	C_p [J/(kg·K)]	*a* [mm^2/s]
Quartile, *Q1*	0.6623	1.7319	915.4	0.3830
Median, *M*	0.6641	1.7334	916.1	0.3836
Quartile, *Q3*	0.6644	1.7343	916.6	0.3836
Interquartile range, *IQR = (Q3 - Q1)*	0.0021	0.0024	1.3	0.0006
Higher outlier, *HO = Q3 + 1.5·IQR*	0.6665	1.7367	917.9	0.3842
Lower outlier, *LO = Q1 - 1.5·IQR*	0.6602	1.7295	914.1	0.3824
Average value, $\bar{X}$	0.6644	1.7330	916.0	0.3834
Standard deviation, *s*	0.0011	0.0014	0.7	0.0007
Coefficient of variation, *CV* [%]	0.16	0.08	0.08	0.18
Upper critical value, *UCV*	0.6655	1.7345	916.8	0.3841
Lower critical value, *LCV*	0.6633	1.7316	915.2	0.3826

Table S8. The obtained values of the thermal parameters of the geopolymer samples with fireclay (1:1.2), and the calculated statistical parameters after 3 days of conditioning in 60°C

Statistical parameters	Thermal properties of sample RSF1.2			
	λ [W/(m·K)]	C_v [MJ/(m^3·K)]	C_p [J/(kg·K)]	*a* [mm^2/s]
Quartile, *Q1*	0.6421	1.6462	875.5	0.3886
Median, *M*	0.6444	1.6528	877.4	0.3913
Quartile, *Q3*	0.6467	1.6534	879.2	0.3935
Interquartile range, *IQR = (Q3 - Q1)*	0.0046	0.0072	3.7	0.0049
Higher outlier, *HO = Q3 + 1.5·IQR*	0.6513	1.6606	882.9	0.3984
Lower outlier, *LO = Q1 - 1.5·IQR*	0.6375	1.6390	871.9	0.3837
Average value, $\bar{X}$	0.6456	1.6508	878.1	0.3911
Standard deviation, *s*	0.0027	0.0036	2.02	0.0023
Coefficient of variation, *CV* [%]	0.42	0.22	0.23	0.58
Upper critical value, *UCV*	0.6485	1.6546	880.2	0.3935
Lower critical value, *LCV*	0.6427	1.6470	876.0	0.3888

Statistical parameters	Thermal properties of sample GF1.2Fe0.5			
	λ [W/(m·K)]	C_v [MJ/(m^3·K)]	C_p [J/(kg·K)]	*a* [mm^2/s]

Quartile, *Q1*	0.6432	1.6408	843.7	0.3919
Median, *M*	0.6435	1.6415	845.0	0.3922
Quartile, *Q3*	0.6440	1.6438	845.5	0.3931
Interquartile range, *IQR = (Q3 - Q1)*	0.0008	0.0030	1.8	0.0012
Higher outlier, *HO = Q3 + 1.5·IQR*	0.6448	1.6468	847.3	0.3943
Lower outlier, *LO = Q1 - 1.5·IQR*	0.6424	1.6378	841.9	0.3907
Average value, $\bar{X}$	0.6442	1.6417	845.3	0.3924
Standard deviation, *s*	0.0006	0.0021	1.0	0.0008
Coefficient of variation, *CV* [%]	0.09	0.13	0.12	0.19
Upper critical value, *UCV*	0.6448	1.6439	846.4	0.3932
Lower critical value, *LCV*	0.6435	1.6395	844.3	0.3916

Statistical parameters	Thermal properties of sample GF1.2Fe1.0			
	λ [W/(m·K)]	C_v [MJ/(m^3·K)]	C_p [J/(kg·K)]	*a* [mm^2/s]
Quartile, *Q1*	0.6662	1.6868	898.4	0.3947
Median, *M*	0.6671	1.6881	899.2	0.3953
Quartile, *Q3*	0.6685	1.6896	900.3	0.3988
Interquartile range, *IQR = (Q3 - Q1)*	0.0023	0.0028	1.9	0.0041
Higher outlier, *HO = Q3 + 1.5·IQR*	0.6708	1.6924	902.2	0.4029
Lower outlier, *LO = Q1 - 1.5·IQR*	0.6639	1.6840	896.5	0.3906
Average value, $\bar{X}$	0.6701	1.6880	899.8	0.3969
Standard deviation, *s*	0.0026	0.0018	0.9	0.0032
Coefficient of variation, *CV* [%]	0.39	0.11	0.10	0.81
Upper critical value, *UCV*	0.6728	1.6899	900.7	0.4003
Lower critical value, *LCV*	0.6673	1.6861	898.8	0.3935

Statistical parameters	Thermal properties of sample GF1.2Fe1.5			
	λ [W/(m·K)]	C_v [MJ/(m^3·K)]	C_p [J/(kg·K)]	*a* [mm^2/s]
Quartile, *Q1*	0.6770	1.6690	854.1	0.4056
Median, *M*	0.6775	1.6703	854.5	0.4059
Quartile, *Q3*	0.6786	1.6709	854.8	0.4064
Interquartile range, *IQR = (Q3 - Q1)*	0.0016	0.0019	0.7	0.0008
Higher outlier, *HO = Q3 + 1.5·IQR*	0.6802	1.6728	855.5	0.4072
Lower outlier, *LO = Q1 - 1.5·IQR*	0.6754	1.6671	853.4	0.4048
Average value, $\bar{X}$	0.6784	1.6702	854.7	0.4062
Standard deviation, *s*	0.0009	0.0011	0.4	0.0009
Coefficient of variation, *CV* [%]	0.13	0.06	0.05	0.23
Upper critical value, *UCV*	0.6793	1.6713	855.2	0.4072
Lower critical value, *LCV*	0.6775	1.6690	854.3	0.4052

Statistical parameters	Thermal properties of sample GF1.2Fe2.0			
	λ [W/(m·K)]	C_v [MJ/(m^3·K)]	C_p [J/(kg·K)]	*a* [mm^2/s]
Quartile, *Q1*	0.6899	1.6955	914.5	0.4101
Median, *M*	0.6955	1.6982	916.0	0.4110
Quartile, *Q3*	0.6975	1.7001	917.0	0.4111
Interquartile range, *IQR = (Q3 - Q1)*	0.0076	0.0046	2.5	0.0010
Higher outlier, *HO = Q3 + 1.5·IQR*	0.7051	1.7047	919.5	0.4121
Lower outlier, *LO = Q1 - 1.5·IQR*	0.6823	1.6909	912.0	0.4091
Average value, $\bar{X}$	0.6958	1.6952	914.3	0.4094
Standard deviation, *s*	0.0032	0.0081	4.4	0.0043
Coefficient of variation, *CV* [%]	0.46	0.48	0.48	1.05
Upper critical value, *UCV*	0.6992	1.7037	918.9	0.4139
Lower critical value, *LCV*	0.6924	1.6867	909.7	0.4048

Statistical parameters	Thermal properties of sample GF1.2Fe2.5			
	λ [W/(m·K)]	C_v [MJ/(m^3·K)]	C_p [J/(kg·K)]	a [mm^2/s]
Quartile, *Q1*	0.6673	1.6550	850.9	0.4040
Median, *M*	0.6685	1.6570	853.4	0.4044
Quartile, *Q3*	0.6715	1.6646	854.5	0.4054
Interquartile range, *IQR = (Q3 - Q1)*	0.0042	0.0096	3.6	0.0014
Higher outlier, *HO = Q3 + 1.5·IQR*	0.6757	1.6742	858.1	0.4068
Lower outlier, *LO = Q1 - 1.5·IQR*	0.6631	1.6454	847.3	0.4026
Average value, $\bar{X}$	0.6716	1.6582	854.7	0.4050
Standard deviation, *s*	0.0023	0.0056	2.6	0.0032
Coefficient of variation, *CV* [%]	0.34	0.34	0.31	0.79
Upper critical value, *UCV*	0.6740	1.6640	857.5	0.4083
Lower critical value, *LCV*	0.6691	1.6523	852.0	0.4017
